# Large-area photonic circuits for terahertz detection and beam profiling

**DOI:** 10.1038/s41377-025-02089-1

**Published:** 2026-01-01

**Authors:** Alessandro Tomasino, Amirhassan Shams-Ansari, Marko Lončar, Ileana-Cristina Benea-Chelmus

**Affiliations:** 1 Hybrid Photonics Laboratory, École Polytechnique Fédérale de Lausanne (EPFL), CH-1015 Lausanne, Switzerland; 2 Center for Quantum Science and Engineering (EPFL), CH-1015 Lausanne, Switzerland; 3 Harvard John A. Paulson School of Engineering and Applied Sciences, Harvard University, Cambridge, MA USA; 4 DRS Daylight Solutions, 16465 Via Esprillo, San Diego, CA USA

**Keywords:** Nonlinear optics, Integrated optics

## Abstract

Deployment of terahertz communication and spectroscopy systems relies on the availability of low-noise and fast detectors, with plug-and-play capabilities. However, most current technologies are stand-alone, discrete components. They are often slow or susceptible to temperature drifts and require tight beam focusing to maximize the signal-to-noise of the detector. Here, we demonstrate an integrated photonic architecture in thin-film lithium niobate that addresses these challenges by exploiting the electro-optic modulation induced by a terahertz signal onto an optical beam at telecom frequencies. Leveraging on the low optical losses provided by this platform, we integrate a double array of up to 18 terahertz antennas within a Mach–Zehnder interferometer, considerably extending the device collection area and boosting the interaction efficiency between the terahertz signal and the optical beam. We show that the double array coherently builds up the probe modulation through a mechanism of quasi-phase-matching, driven by a periodic terahertz near-field pattern, circumventing physical inversion of the crystallographic domains. This provides means to fully custom-tailor the frequency response of the device, limit it to a desired frequency band and effectively suppress out-of-band signals. The large detection area ensures correct operation with diverse terahertz beam settings. Furthermore, we show that the antennas act as pixels that allow reconstruction of the terahertz beam profile impinging on the detector area. Our on-chip design in thin-film lithium niobate overcomes the detrimental effects of two-photon absorption and fixed phase-matching conditions, which have plagued previously explored electro-optic detection systems, especially in the telecom band, paving the way for more advanced on-chip terahertz systems.

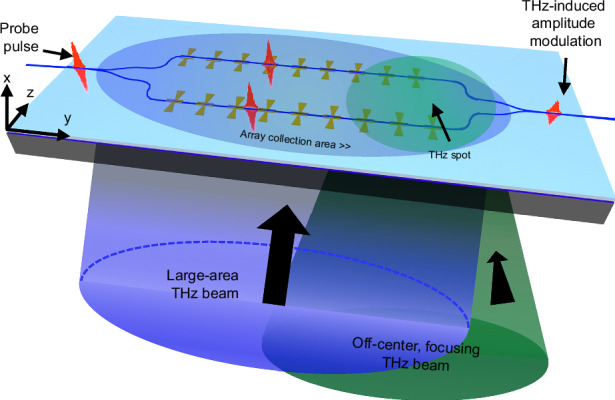

## Abstract (web summary)

We demonstrate a large-area, integrated photonic architecture in thin-film lithium niobate relying on the electro-optic modulation of a telecom beam to perform field-resolved terahertz detection and beam profiling

## Introduction

The ever-growing demand for applications such as artificial intelligence, augmented reality, internet of things, wireless communication, and cloud-based computing is requiring ever-increasing bandwidths and frequency availability, pushing the next generation of communication systems (namely, 6G^1^) to utilize unprecedentedly high carrier frequencies, largely exceeding 100 GHz^2–4^. The terahertz (THz) spectral range, with frequencies conventionally between 0.1 and 10 THz^5^, bridges the microwaves and optical domains and emerges as a valuable resource towards realizing these high-speed and high-fidelity communication channels^6,7^. Compared to optical beams, THz radiation exhibits a much longer wavelength, thereby being much less affected by Mie scattering^8^ and atmospheric scintillations (i.e., random fluctuations in the refractive index of the atmosphere^9^). This allows for the realization of communication channels that are more robust against environmental disturbances^10–12^. On the other hand, compared to microwaves, free-propagating THz beams are significantly more directional, and are of interest for point-to-point communications. However, the generation and detection of THz waves are often implemented through very high-gain antennas to compensate for the severe free-path loss occurring within the low atmosphere^13^. Narrower beams allow for more accurate illuminations of the desired target, implying a more efficient use of the THz power and a reduced risk of eavesdropper attacks^14^. The higher directionality of THz waves demands developing a new set of hardware capable of keeping receivers oriented towards the THz communication link, especially in the case of non-stationary access points^15–17^. As such, the reliable and widespread adoption of THz technology necessitates versatile THz wireless detectors that maintain high sensitivity for signals either collimated or focused^18^. Current THz sensing technologies include bolometers^19–21^, graphene-based detectors (both for electronic and optical read-out)^22–25^, Golay cells^26^ and pyroelectric detectors^27,28^. These types of detectors are not easily compatible with available communication and sensing infrastructures due to practical limitations, such as cryogenic operating temperatures, relatively high dark currents, and long recovery times. More importantly, their incoherent nature, i.e., being sensitive to the THz intensity rather than the THz electric field, results in the loss of the phase information.

Coherent detectors are particularly important in communications and sensing, providing access to both the amplitude and phase of the THz electric field. Common phase-sensitive detection schemes are based on the coherent up-conversion of THz signals to the optical domain, using either photoconductive switches (i.e., photomixer)^29,30^ or the electro-optic effect in bulk crystals exhibiting a second-order nonlinearity. In the latter case, the THz field induces an amplitude-dependent polarization modulation on a probe pulse via frequency up-conversion. These detection schemes operate at room temperature and most importantly provide very low-noise readouts^31,32^.

**Fig. 1:**
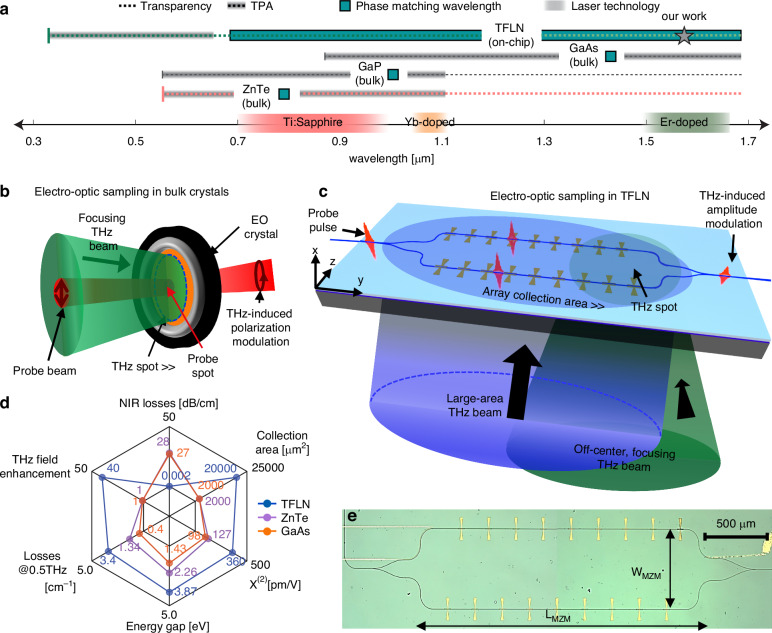
**Electro-optic sampling in bulk crystals versus on-chip in thin-film lithium niobate**. **a** Available material platforms for THz transient detection using the electro-optic sampling technique. Due to their inherent dispersion properties, the phase-matching wavelength is fixed for bulk electro-optic (EO) crystals, such as ZnTe, GaAs, and GaP. The bottom axis shows the most widespread laser technologies used for electro-optic sampling, which do not always provide emission at the exact phase-matched wavelengths (see GaAs). Thin-film lithium niobate (TFLN) provides means to engineer group and phase velocities at both THz and optical frequencies, enabling phase-matching in a rather continuous optical range. Moreover, thanks to its large bandgap, TFLN allows for THz detection far from wavelength ranges affected by two-photon absorption (TPA). This is especially advantageous in the telecommunication band, where fiber optic infrastructure is available, whereas crystals such as GaAs are impractical due to TPA. **b** Conventional implementation of electro-optic sampling in bulk crystals. Both the optical and the THz beams are tightly focused onto the EO-crystal. The THz beam induces a field-dependent polarization modulation on the probe beam. The latter is measured via ellipsometry (not shown in the figure). In this approach, a great limitation to the THz signal-optical probe interaction is the large mismatch between the THz and optical spot sizes. **c** Implementation of electro-optic sampling in waveguide-based TFLN platform. This approach relies on the interaction of the probe pulse with an array of THz antennas deposited on each arm of an interferometer on-chip. The incident THz beam generates a phase modulation on the probe beam traveling through each arm, leading to the THz-induced amplitude modulation of the probe beam at the output of the interferometer. The antenna arrays effectively increase the sensitive area of the detector to a physical footprint of a few millimeters, extending the use of this type of device to operate with both large area and off-center THz beams. **d** Radar chart reporting a comparison among the main THz-detection platform properties for the cases of TFLN (blue), ZnTe (purple), and GaAs (orange). Values represented by circles are data taken from refs. ^37^ and^75^ for ZnTe, from refs. ^76^ and^77^ for GaAs, and from refs. ^49^ and^78^ for TFLN. Each axis (i.e., hexagon diagonal) is in linear scale. The inner black hexagon represents the zero value for each numerical crystal parameter, whereas numbers in black on the outer hexagon represent upper boundaries used for references. TFLN supersedes the other platforms in THz field enhancement, TPA, optical losses, sensitive area, and nonlinearity. Terahertz losses are greater for bulk LN, yet TFLN technology mitigates this effect owing to the deep sub-wavelength size of the waveguides compared to the THz wavelength. **e** Optical micrograph of the Mach Zehnder interferometer realized in TFLN, with a length of *L*_*MZM*_ = 2.6 mm and a width of *W*_*MZM*_ = 670 μm

However, commonly used crystals such as zinc telluride^33–36^, gallium arsenide and gallium phosphide^37^ operate at precise phase-matching wavelengths, due to their inherent dispersion relations in both the optical and terahertz domain (Fig. 1a). Due to the relatively narrow spectral range over which phase matching is possible, the wavelength of the probe beam needs to be precisely chosen according to the properties of the crystal at THz frequencies. As a result, the emission wavelength of the available laser technologies (such as, Ti:Sapphire and Yb:YAG) need to be compatible with such crystals. This constraint may be relaxed with thin crystals, but this reduces the total interaction length between the THz and the optical beam. Achieving phase matching over extended optical frequencies would therefore provide greater flexibility in the design of THz systems. A further complication is that the phase-matching wavelength often overlaps with the range of two-photon absorption, resulting in severe nonlinear absorption and thus limiting the maximum applicable optical power. Finally, their spectral response is often broadband, which is undesired in communication links that aim to suppress out-of band interference. In these free-space electro-optic detection schemes (Fig. 1b), a maximal modulation is achieved by strongly focusing the THz beam, ideally down to the diffraction limit. Owing to the up-conversion mechanism, the THz detection relies on detecting the polarization modulation via ellipsometry using commercially available optical detectors.

However, since the THz spot is often much larger than the probe, the THz energy is typically not efficiently converted to the optical domain. A potential solution for controlling the frequency response, phase-matching wavelength and simultaneously address shortcomings of bulk systems is transitioning these technologies to on-chip^38–40^. For instance, integrated antennas allow for simultaneously targeting a specific THz frequency range where the performance is optimized and achieving strong field enhancement beyond the diffraction limit^41^. This is in contrast with THz systems based on bulk crystals, where the sensitive bandwidth is dictated by the phase-matching condition. In previous demonstrations, THz antennas were integrated with chip-based plasmonic waveguides based on organic electro-optic molecules^42–44^. Despite the large second-order nonlinearity of the organic molecules, plasmonic approaches suffer from large propagation losses of (0.25 dB μm^−1^), which prevents the integration of multiple antennas so as to form a long sequence, thus hindering the realization of large photonic circuits. As a result, those devices were hampered by an inefficient collection of the THz power due to the large mismatch between the THz spot and the antenna collection area. Furthermore, the silicon-based integrated circuits suffer from large two-photon absorption, thus limiting the on-chip optical probe power and resulting in poor signal-to-noise ratio of the electro-optic detection^45^.

Given these constraints, it is imperative to develop strategies that enable an improved collection and up-conversion of millimeter-sized THz waves, and a customizable spectral response. Among available integrated photonics platforms, thin-film lithium niobate (TFLN) is particularly well suited, as it can achieve phase-matching outside the two-photon absorption range, for example at 1550 nm where fiber technologies are well developed^46–48^. Here, we show that this property is essential for the realization of a large area photonic circuit that implements a quasi-phase matching mechanism using antenna arrays to achieve efficient phase-sensitive terahertz detectors with a fully customizable frequency response. We exploit the low propagation losses of 1.3 dB m^−1^^49^ and absorption-limited loss of 0.2 dB m^−1^^50^, along with the high Pockels coefficient (*r*_33_ ≈ 30.9 pm V^−1^^51^) of lithium niobate (LN), to realize a THz detector operating at a nominal frequency of 0.5 THz (Fig. 1c), implemented in a millimeters-long Mach–Zehnder interferometer (MZI). The THz wave imparts a phase modulation to the optical probe via the electro-optic (Pockels) effect while being collected from an increased area defined by an array of 18 gold bow-tie antennas, each providing a few tens-fold enhancement of the THz field. An appropriately chosen distance between individual antennas allows for operations according to a mechanism similar to quasi-phase-matching, which extends the nonlinear interaction between the THz and optical probe beams, resulting in a coherent build-up of the probe modulation across the entire array. Our TFLN platform provides combined merits of up to a 30-fold larger THz field enhancement, 100 times larger collection area, 3–5 times larger *χ*^(2)^ (for THz-optical nonlinear interactions), 30 times lower linear loss of the telecom beam and twice larger energy gap compared to routinely used bulk crystals (summarized in Fig. 1d). Furthermore, the nanoscale design allows us to partially mitigate the approximately twice higher loss of lithium niobate compared to zinc telluride. Altogether, these properties enable peak-to-peak time-domain modulation efficiencies exceeding *η* = 0.8 × 10^−3^, obtained under the illumination of a test THz pulse with a field strength of ~4.5 V cm^−1^. Moreover, the phased array provides significant spectral sensitivity around the operating frequency, with a minimum linewidth of 46 GHz and up to 40-dB out of band suppression, maintaining comparable performance even when subjected to off-center and out-of-focus THz beam illuminations. Finally, the large area of the detector enables efficient mapping of the THz beam profile through a single one-dimensional (1D) scan. This capability opens avenues for applications in radar systems and target-locking mechanisms for moving objects.

## Results

### Geometry and properties of the large area terahertz detector

A schematic of our Mach–Zehnder interferometer-based detector and an optical microscope image of its top view is depicted in Fig. 1c and e, respectively. A series of THz antennas is realized by patterning the metallic contacts on both sides of a waveguide fabricated on an x-cut TFLN wafer. Such antennas allow for coupling the free-space THz radiation onto the chip (fabrication details of our devices are provided in Methods, whereas the exact geometric dimensions are listed in Supplementary Note 1). An optical probe beam traveling inside the TFLN waveguide splits into two at the input y-splitter of the interferometer. Our design aligns the optical mode polarization to the crystallographic z-axis of the TFLN waveguide, thus exploiting the *r*_33_ Pockels coefficient^52^ - the largest available in a waveguide geometry (see the optical mode simulations in Supplementary Note 1). The THz beam is incident orthogonally from the backside of the high-resistivity silicon substrate and simultaneously illuminates all bow-tie antennas, leading to their synchronous resonance. The incident THz radiation is polarized along the crystallographic axis of lithium niobate (z-axis), which also coincides with the orientation of the bow-tie antennas, thus simultaneously maximizing their collection efficiency (Fig. 1c) and the nonlinear interaction. After crossing the arms of the interferometer, the two probes are recombined at the output y-splitter and then sent to an infrared photodetector for acquisition.

### Quasi phase-matching with terahertz antenna arrays

**Fig. 2:**
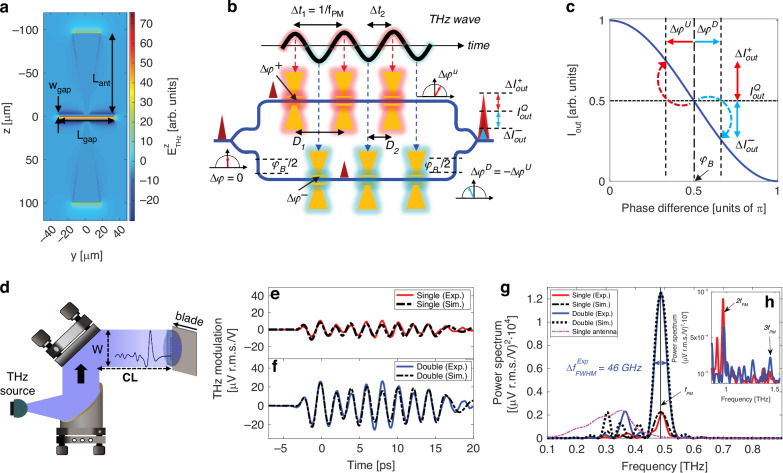
**Quasi-phase-matching with antenna arrays**. **a** Simulation of the enhanced THz electric field on the z-y plane of the antenna, at the resonance, computed via CST Microwave Studio. The field is uniformly distributed along the entire gap ensuring coherent build-up of the probe phase modulation. For the sake of clarity, the geometrical parameters of the antenna are overlaid to the electric field map. **b** Sketch depicting the coherent build-up of the phase modulation imparted to the probe beam by the THz wave and leading to the amplitude modulation operated by the MZI. The latter is realized with an arm length difference leading to a build-in phase imbalance (*ϕ*_*B*_) allowing for operation at its quadrature point. A probe beam with an initially null THz-induced phase retardation (Δ*ϕ* = 0) enters the MZI and is split into two identical beams. Each probe crosses an array of *N*_*ant*_ antennas (shown in number of 3 for simplicity), where it experiences a phase retardation that is proportional to the THz electric field established in the antenna gap. The spatial period of the array (*D*_1_) sets the arrival of the probe beam at each antenna at multiples of the time interval Δ*t*_1_. This leads to a coherent build-up of all phase modulation contributions imparted along the entire array. If the lower array is displaced by a distance *D*_2_ (corresponding to a time interval Δ*t*_2_), the probe beam in the lower arm will cross each antenna when the THz field oscillations exhibit an opposite polarity compared to the top arm. The latter will impart a total phase modulation of a reverse sign, interfering with that of the top arm and leading to the intensity modulation of the probe beam at the output of the MZI. **c** Transmission curve of the interferometer operating at the quadrature point excited by a bipolar THz wave where the phase modulation Δ*ϕ*^*U*^/Δ*ϕ*^*D*^ changes the output probe intensity by $\Delta {I}_{out}^{+}/\Delta {I}_{out}^{-}$. The plot is not to scale, and deviations from the quadrature point are exaggerated to better visualize the operating principle. In our experiment, relative amplitude variations accounts for less than 0.1%, thus ensuring a linear response of the device a function of the driving THz field, see text. **d** Experimental configuration to demonstrate the phase-matching mechanism. The broadband THz beam used to study the impulse response of the device *H*(*f*), is collimated with a diameter of W = 4 cm and then travels for CL = 57 cm in free space before reaching the chip. To test the case of the single-arm illumination, half of the interferometer is covered with a metallic blade. **e** THz electric field waveforms reconstructed for the case of single arm illumination (red solid line) and **f** double arm illumination (blue solid line). In both panels the black dash/dotted lines represent the results of the analytical model computed via Eq. (1), using the simulated complex field *E*_*ant*_(*f*) as described in the main text. **g** Power spectra obtained by Fourier transform of the waveforms in (**e**) and (**f**), for both experimental and simulated cases. The full-width half-maximum linewidth $\Delta {f}_{FWHM}^{Exp}$ retrieved for the double arm illumination shows an excellent agreement with that calculated analytically. For comparison, we show the spectrum experimentally retrieved with a MZI device hosting a single antenna on only one of its arm (pink dot-dashed line). **h** zoom-out of plot in (**g**) showing higher harmonics, namely second (2*f*_*PM*_) and third (3*f*_*PM*_)

Our detector is designed to operate at a desired THz frequency *f*_*PM*_ (phase matching frequency) and exhibit a suppressed response away from this frequency, in contrast to other terahertz detectors that target broadband operation. Both the individual antenna and array configuration are designed in order to maximize the cumulative modulation of the probe beam induced by the impinging THz signal. Each antenna features a pair of elongated strip conductors (with a length *L*_*gap*_)^53^, which prolong the THz-probe interaction and improve the efficiency of the nonlinear interaction, in contrast to more conventional designs^54–56^. Figure 2a shows the THz electric field established across the z-y plane of the antenna, revealing a homogeneous field distribution along the gap length, with a 60-fold and 30-fold field enhancement near the gold strip conductors and in the center of the gap, respectively. The probe beam propagates through the gap within a time interval *τ*_*gap*_ = *L*_*gap*_*n*_*g*_/*c*, where *n*_*g*_ is the optical group index and *c* is the speed of light in vacuum. By choosing a gap length much shorter than a THz wavelength (e.g., *L*_*gap*_ ≈ *λ*_*T*_/10), the probe beam will take a fraction of the duration of a complete THz cycle to cross the entire gap length, i.e. *τ*_*gap*_ ~ 1/(10*f*_*T*_), with *f*_*T*_ = *c*/*λ*_*T*_ being the resonant frequency of the antenna. This ensures that the phase modulation imparted by each antenna builds up constructively along its entire gap^42,43^, accounting for a contribution $\Delta \phi (t)\propto {r}_{33}{E}_{{\rm{THz}}}^{ant}(t)$, linearly dependent on the instantaneous THz electric near-field ${E}_{{\rm{THz}}}^{ant}(t)$. In a geometry where the antennas resonate synchronously, the probe beam encounters the n-*th* antenna at the time instant *t*_*n*_, thus experiencing a phase delay Δ*ϕ*_*n*_ ∝ *r*_33_*E*_THz_(*t*_*n*_). The total phase modulation accumulated at the end of the series is then the sum over all *N*_*ant*_ contributions, i.e., $\Delta {\phi }_{tot}=\mathop{\sum }\nolimits_{n = 1}^{{N}_{{\rm{ant}}}}\Delta {\phi }_{n}$. If the time instants *t*_*n*_ are all different, Δ*ϕ*_*tot*_ may be much lower than that of a single antenna. Therefore, in order to effectively benefit from the array configuration, it is crucial to engineer its periodicity to allow for a coherent adding-up of all Δ*ϕ*_*n*_ at the design frequency *f*_*PM*_, thus largely exceeding the value of the single antenna element. This mechanism is analogous to quasi-phase matching^57–59^, yet does not require performing periodic poling of the chip (Fig. 2b). Along each arm of the interferometer, we designed the distance between two consecutive antennas (*D*_1_) in such a way that the oscillation period of the THz electric field within their gap matches the arrival time of the probe beam at each antenna. This synchronization ensures that the probe beam acquires identical phase modulation contributions Δ*ϕ*^+^ while crossing each antenna along the array, resulting in a total phase modulation of Δ*ϕ*^*U*^ = *N*_ant_Δ*ϕ*^+^ for the upper arm. The distance between two consecutive antennas *D*_1_ defines the phase-matching frequency ${f}_{{\rm{PM}}}=\frac{1}{\Delta {t}_{1}}$ of the array, with $\Delta {t}_{1}=\frac{{n}_{g}{D}_{1}}{c}$ the group delay of the optical probe between two consecutive antennas. In the simple case when only arm of the MZI hosts an array of antennas, the interference with its unmodulated counterpart traveling through the plain arm generates an interference that converts the acquired phase modulation of the single arm into the amplitude modulation of the resulting output beam. This allows for the use of a simple photodiode to reconstruct the THz signal. In order to further boost the collection efficiency of the MZI and benefit from a push-pull effect where the lower and upper arm contribute equally to the total phase modulation, a second array of the antennas is placed on the lower arm. However, such a second array cannot mirror the position of that on the upper arm, because it would give rise to the exact same THz-induced phase modulation, preventing the desired amplitude modulation at the output of the interferometer (see Supplementary Note 4). To circumvent this issue, the second array is displaced by a distance *D*_2_ = *D*_1_/2 (Fig. 2a) with respect to the upper one. Such a displacement causes the lower probe to lag behind the upper one by a time delay $\Delta {t}_{2}=\frac{\Delta {t}_{1}}{2}=\frac{1}{2{f}_{{\rm{PM}}}}$. As a result, the probe beam crosses the antennas of the lower array during the negative half cycle of the THz near-field oscillations, resulting in a phase retardation contribution Δ*ϕ*^−^ of an opposite sign compared to the upper arm. Consequently, the lower probe will build up a total phase modulation with a reversed sign Δ*ϕ*^*D*^ = *N*_ant_Δ*ϕ*^−^ = − *N*_ant_Δ*ϕ*^+^.

The two arms of the interferometer are designed with slightly different optical path lengths so that the built-in phase difference *ϕ*_*B*_ for the telecom probe is equal to *π*/2. Under this condition, the interferometer operates in the so-called quadrature point, providing an unmodulated output intensity ${I}_{out}^{Q}$ that is half of that at the maximum transmission ${I}_{out}^{0}$ as indicated in Fig. 2c. On this point, the curve slope $d{I}_{out}/d\phi ={I}_{out}^{0}/2$ exhibits a maximum, thus allowing for the highest sensitivity to a phase change, and consequently to the incident THz electric field. Any additional phase imbalance Δ*ϕ*^*U*^ (or Δ*ϕ*^*D*^) due to the THz-induced Pockels effect, changes the output intensity by $\Delta {I}_{out}^{+}$ (or $\Delta {I}_{out}^{-}$). For small values of the total phase modulation, i.e., Δ*ϕ*_*tot*_ ≪ 1, the relative intensity modulation is directly proportional to driving THz electric field *E*_*THz*_, i.e., $\Delta {I}_{out}/{I}_{out}^{Q}\propto (\Delta {\phi }^{U}-\Delta {\phi }^{D})\propto {E}_{THz}$^43^. In order to demonstrate the frequency selectivity and the quasi-phase matching mechanism of the interferometer, we derived an analytical expression describing the frequency response of our detector in terms of THz-induced variations of the probe intensity Δ*I*(*f*) (see Supplementary Note 4 for a detailed derivation):

1 $$\frac{\Delta I(f)}{{I}_{out}^{Q}}\propto \Delta {\phi }^{U}-\Delta {\phi }^{D}=-2i{E}_{ant}(f)\frac{\sin (\pi f{N}_{ant}\Delta {t}_{1})}{\sin (\pi f\Delta {t}_{1})}\sin (\pi f\Delta {t}_{2}){e}^{i\pi f\Delta {t}_{1}({N}_{ant}-1)}{e}^{i\pi f\Delta {t}_{2}}$$

where *f* is the THz frequency and *E*_*ant*_(*f*) is the THz electric near-field established inside the antenna gap, assuming a uniform THz illumination across the entire device. Equation (1) provides guidelines to realize a detector sensitive at a desired frequency with a chosen bandwidth. In the specific case that Δ*t*_2_ = Δ*t*_1_/2 = Δ*t*/2, Eq. (1) becomes:

2 $$\frac{\Delta I(\,f)}{{I}_{out}^{Q}{E}_{ant}(\,f)}\propto {T}_{MZI}(\,f)=-2i\frac{\sin (\pi f{N}_{ant}\Delta t)}{\sin (\pi f\Delta t)}\sin \frac{\pi f\Delta t}{2}{e}^{i\pi f\Delta t({N}_{ant}-1/2)}$$

where we have introduced *T*_*MZI*_(*f*), defined as the complex spectral response of the device. In Supplementary Note 4 we demonstrate that a higher number of antennas inversely decreases the detector bandwidth. At the same time, the amplitude response quadratically increases as more antennas are added to the interferometer arms. This outcome is in line with the quasi-phase-matching theory.

To experimentally demonstrate the proposed quasi-phase-matching mechanism, we retrieved the impulse response *H*(*f*) ≡ ∣*T*_*MZI*_(*f*)∣ of our detector via illumination with a collimated broadband THz beam (Fig. 2d). The use of such a test THz pulse, sufficiently short to act as a delta function, allows us to reconstruct both its time- and frequency-domain responses, thus highlighting the key differences with flat-band detection schemes, such as the free-space electro-optic sampling technique (see Supplementary Note 6 for a detailed comparison). Specifically, to record the temporal response, we performed on-chip electro-optic sampling using femtosecond laser pulses as optical probes (full measurement details are given in Methods and Supplementary Note 2). We generated phase-locked THz pulses via a photoconductive antenna excited by a femtosecond near-infrared laser. We then acquired the intensity of the out-coupled probe beam by scanning the mutual delay between the arrival times of the THz and probe pulses in the chip using a mechanical delay line. This allows for the acquisition of a time-varying waveform reproducing the temporal response of our detector. The collimated THz beam test had a diameter of W = 4 cm, propagating in free space for a distance of CL = 57 cm before reaching our chip, thus respecting the assumption underlying Eq. (1). We further verified the performance of our device when illuminating just the antenna array in the upper arm (Fig. 2e) or the arrays in both the upper and lower arm (Fig. 2f), by covering half of the interferometer with a metallic blade (Fig. 2d). We report the measured THz transients in units of absolute amplitude modulation Δ*V*/*V* of the electrical signal acquired from the photodiode to back-track the modulation efficiency. We observe that the amplitude of the signal retrieved for the fully illuminated double array (f) is twice larger than that of the singly-illuminated array (e), confirming the outcomes of our analytical model. To reproduce the experimental temporal response of the device, we simulated the broadband THz electric near-field established in the antenna gap *E*_*ant*_(*f*) and included it into the analytical expression of Eq. (2). More details are provided in the Supplementary Note 4. This way, we experimentally confirmed that the fundamental frequency *f*_*PM*_ is highly enhanced for the case of the fully illuminated double array, with a phase-matching frequency of 487 GHz and a linewidth of only 46 GHz, in excellent agreement with simulation results predicting 487 GHz and 48 GHz, respectively (see Fig. 2e for comparison). Finally, we observe in the inset of Fig. 2g, that the frequency component at 2*f*_*PM*_ is much stronger for the case of the single array, while the opposite occurs for the third harmonic at 3*f*_*PM*_, demonstrating clear suppression of even components in the double array case. These experimental findings demonstrate that our design has the ability to enhance the responsivity of a THz detector in a desired band spanning a few tens of GHz while suppressing its out-of-band response by 20–40 dB, depending on frequency (see Supplementary Note 6). This capability is beneficial for ensuring large data bandwidths and reducing cross-talk in communication systems.

### Operation under off-center illumination and beam profiling

**Fig. 3:**
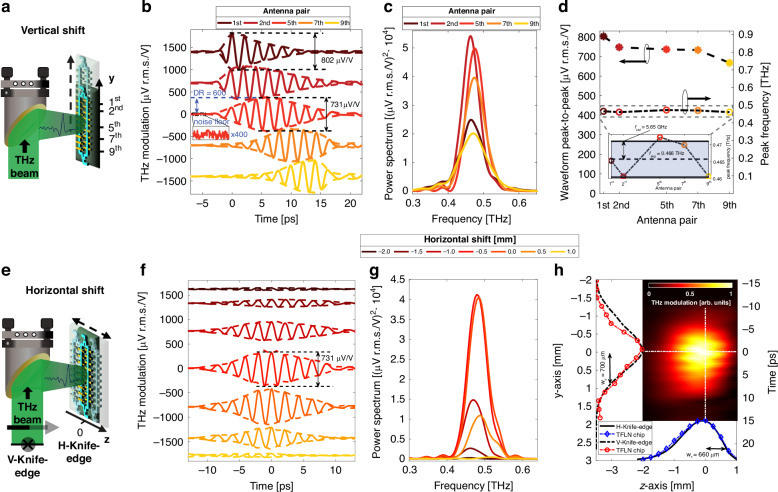
**Large area TFLN circuits for waveform detection and beam profiling in the focal plane**. **a** Experimental configuration for measuring the impulse response of the TFLN chip to a broadband THz pulse test, at different positions of the THz spot along the antenna array (y-axis). **b** Terahertz waveforms recorded for various values of vertical shifts (solid lines) and fitted envelopes (dashed lines). The waveforms share similar peak-to-peak values as well as dynamic ranges (DR) of about 600 at 300 ms integration time per point, calculated as the ratio between the waveform peak and the corresponding noise floor (recorded at time instants preceding the THz signal and magnified by 400 times in the inset). The position of the peak of the envelope coincides with the pair of antennas that are spatially aligned with the THz beam. The maximum THz modulation remains relatively constant across all measurements. All curves are vertically separated for clarity. **c** Calculated power spectra of the waveforms in (**b**), showing larger spectral amplitudes at *f*_*PM*_ for the case where the THz spot is aligned with the center of the antenna array. **d** Terahertz modulation peak (stars) and peak frequency (open circle) show little dependence on any specific antenna pairs under illumination. A zoom-in of peak frequency behavior upon horizontal shifts is given in the inset in (**d**), showing that the operating frequency only varies by a few percent of the average value. **e** Experimental configuration for measuring the TFLN chip impulse response to a broadband THz pulse test, as a function of the horizontal shift (z-axis) and to reconstruct the beam profile at the chip. **f** Terahertz waveforms recorded for various values of horizontal shifts (solid lines) and fitted envelopes (dashed lines). The position of the peak of the envelope and the maximum THz modulation remains relatively constant similar to (**b**). Curves are vertically separated for clarity. **g** Calculated power spectra of the waveforms in (**f**). **h** Two-dimensional image (z-y plane) of the focused terahertz spot reconstructed combining the waveforms shown in (**f**). Cut-lines at z = 0 (red circles) and y = 0 (blue diamonds) overlaid with curves profiles (black dotted/dashed line) retrieved via knife-edge measurements

In many applications, another desirable feature for a detector is the capability of functioning under diverse illumination conditions and potentially performing an auto-correction of the device illumination to maintain optimum performance^60^. Specifically, a THz detector should provide an adequate response even when the impinging THz wave is off-centered. To verify this capability, we studied the performance of our device as it moves away from the focal point. This is in contrast to the collimated illumination discussed in the previous section. We vertically shifted the device along the length of the interferometer (y-axis) as sketched in Fig. 3a. The THz spot covers an area of around 660 × 700 μm^2^, determined using a time-domain knife-edge measurement (see Supplementary Note 3), thus being smaller than the entire interferometer footprint.

In fact, using conventional relations for RF Mach Zehnder modulators^61^, we estimate that impinging THz electric fields greater than 10 kV cm^−1^ are required to drive the interferometer to operate across the nonlinear section of its transmission curve. Remarkably, the dynamic ranges, calculated as the ratio between the waveform peak and the standard deviation of the noise floor^62^ are all around DR = 600 for an integration time of 300 ms applied to each point on the waveform (see Fig. 3a). This figure of merit allows us to estimate that the minimum detectable THz signal amplitude impinging on the device is around 7.5 mV cm^−1^, under these measurement settings. Higher sensitivities can be achieved with longer integration times. Moreover, the similar dynamic ranges retrieved for each position of the device prove its robustness against misalignment.

We note that the peak of the envelope shifts from earlier towards later time delays as we move the chip upwards. Moreover, the number of cycles within the envelope depends on the position of the THz spot, indicating an incomplete illumination for positions too far off the center. When the THz beam is centered on the most peripheral antenna pairs (the 1^st^ and 9^th^, respectively), roughly only half of the beam illuminates the array, resulting in an asymmetric envelope. However, as the THz spot moves towards more central positions along the array, a larger number of antennas are efficiently excited. Consequently, the recorded waveform becomes more symmetric with a longer duration of its envelope. The spectral amplitude at the *f*_*PM*_ = 0.487 THz component increases in value as the THz cross-section occupies more central positions, as visible from the power spectra in Fig. 3c calculated via the Fourier-Transform of the curves in Fig. 3b. Finally, we observe in Fig. 3d that our device allows us to maintain a similar modulation peak frequency (within a deviation smaller than 6 GHz), regardless of which and how many antennas are illuminated.

These observations provide insights into the temporal response and capabilities of our large-area detector. A modulation peak invariant with the position indicates that the time response of each antenna lasts only a few cycles, exhibiting a relatively fast decay. Because of this, when the probe beam crosses each antenna at the time instant corresponding to the peak of the THz near-field, the phase modulation due to previous antenna encounters is negligible. Therefore, we conclude that the instantaneous values of the reconstructed THz waveforms are always due to contributions from a single antenna. This effect is here achieved by choosing antennas with low quality factors and resonances significantly different in value from the phase-matching frequency (*f*_*PM*_ = 487 GHz and *f*_*ant*_ = 360 GHz, see Fig. 2g). This effect is especially apparent while comparing the THz transients acquired from central and peripheral illumination. Conversely, if the antennas had a relatively high quality factor (i.e., featuring long-lasting oscillations), the amplitude of the resulting waveform would keep growing with the number of antennas illuminated by the THz wave.

We took advantage of the fast response of the antennas to reconstruct the profile of the THz beam illuminating the chip, using the pairs of antennas as pixels. This is possible because the chosen spacing between antennas allows them to have collection areas that are not fully overlapped. Consequently, the near-field established within the gap is mainly dependent on the THz electric field locally captured by each antenna, and hence can be directly linked to the beam section just above the plane of the antenna. The latter is encoded in the amplitude of the various cycles composing the complete waveform, each of them uniquely associated to specific antenna pairs along the array. We now use the envelope of these waveforms to reconstruct a two-dimensional beam profile of the THz spot at the TFLN chip. As shown in Fig. 3h, this is achieved by mapping the time axis of panel Fig. 3f to a spatial position using the group velocity of the probe beam (*v*_*g*_ = *c*/*n*_*g*_). The THz spot image reconstructed through our chip unveils a non-centered, elliptical shape. Cut-lines at *y* = 0 and *z* = 0 reported in the two insets of Fig. 3h are accurately reproduced by beam profile measurements obtained via time-domain knife-edge measurements (see Supplementary Note 3). This showcases the capability of TFLN circuits to be used for THz beam profiling, providing an adequate dynamic range even at significantly low THz field strengths.

### Operation under out-of-focus terahertz beams

**Fig. 4:**
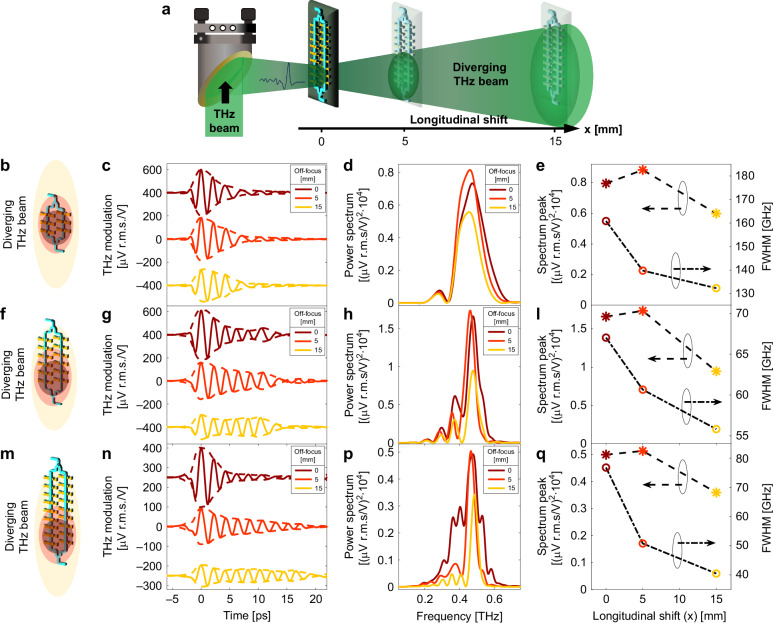
**Detection under off-centered illumination for increasingly long antenna arrays**. **a** Experimental configuration for detecting a diverging THz beam test. The device is initially placed on the THz focal plane, where the THz spot mainly illuminates the initial pairs of antennas. The device is then moved out of the focal plane on the z-axis. Time-domain acquisitions have been carried out at positions x = 0, 5, and 15 mm, away from the focal plane. Three different detector types have been used, featuring **b** 3, **f** 6, and **m** 9 antennas per array. Time traces are reported in panel (**c**, **g**, **n**) for the above-mentioned devices, whereas (**d**, **h**, **p**) shows the corresponding Fourier-Transform calculated spectra. The general trend among all cases is that the THz transients acquire more cycles as the detectors get farther from the focal plane, due to the broadening THz illumination, which covers an increasing number of antennas. Besides, all spectra reveal that at the intermediate z-position of 5 mm, the peak frequency exhibits a higher amplitude, due to an augmented contribution from more antennas, despite the decrease in electric field strength of the diffracting THz beam, compared to the focal plane. These considerations are summarized in plots (**e**, **l**, **q**), which show the trends of the frequency peak and its linewidth as a function of the longitudinal shift. In panels (**c**, **g**, **n**), curves are vertically shifted for clarity

Various terahertz applications in spectroscopy and communications typically require well-defined central frequency and bandwidth. Moreover, maintaining these characteristics across a wide range of illuminations is equally important. Here, we investigate to which extent the proposed quasi-phase-matching mechanism exhibits resilience to off-focused illumination. Specifically, we characterized a series of detectors operating at the same phase-matching frequency *f*_*PM*_ = 487 GHz, yet featuring different numbers of antennas per array, i.e., 3, 6, and 9. This selection allows for direct control over the detection bandwidth using simple lithographic techniques, ensuring high reproducibility and robustness. The detector arrays were placed at three locations, i.e., 0, 5, and 15 mm away from the focal plane so as to be illuminated by a diverging THz beam, as sketched in Fig. 4a. By applying Rayleigh’s law of diffraction for the THz wave, we calculated that the major axis of the THz spot (around 0.7 mm) expanded to a diameter of 4.2 mm and 10 mm after the 5-mm and 15-mm propagating distances, respectively. This is sufficient to cover the entire array length on any device. Because of the reduced size of the 3-antennas detector (Fig. 4b), we aligned its center with the THz spot and then kept the same illumination condition for the other two cases, as depicted in Fig. 4f, m.

The THz transients recorded for each detector type are shown in Fig. 4c, g, n for an increasing number of antennas, respectively. Since the arrays on the 3-antenna device are shorter than 1 mm across, the THz beam covers a great area of the detector already at the focal plane. As such, the corresponding waveforms do not significantly differ in terms of the number of cycles, whereas the transient amplitude decreases for larger longitudinal shifts along the THz path. This is due to THz beam diffraction resulting in a weaker THz electric field intensity. We note that in the frequency domain (Fig. 4d) the spectral amplitude at *f*_*PM*_ first increases and then decreases, as the THz beam diverges (Fig. 4d, h, p). Here, both spectrum peak and linewidth associated with *f*_*PM*_ are plotted as a function of the longitudinal shift (x-axis). While the THz modulation is the highest for intermediate THz beam sizes, the linewidth monotonically diminishes for increasing THz beam size covering the array. These results suggest that there is an optimum THz illumination condition for the operation of the devices in terms of spectral response, as a result of two concurrent effects: field enhancement within the single antenna and additive contributions from a larger number of them. Finally, we observe a quite similar behavior for the 6- and 9-antenna devices, as depicted in Fig. 4g, h and n, p, respectively, with some noticeable differences. The transient duration considerably changes for larger longitudinal shifts, especially for the 9-antenna case, since the initial THz illumination only partially excites all the antennas. This also leads to a faster decrease of the associated linewidth, which shrinks down to around ~40 GHz for the 9-antenna device, 15 mm away from the focal plane (Fig. 4q).

## Discussion

In summary, we demonstrated a coherent THz detector (i.e., measuring both phase and amplitude of the incident field) relying on the Pockels effect in TFLN photonic circuits, operating at a nominal frequency of 500 GHz. Compared to state-of-the-art integrated terahertz detectors, the low optical losses of our platform enable the realization of a Mach–Zehnder interferometer hosting arrays of up to 9 antennas (on each arm) to effectively collect THz signals from free space. This is achieved by exploiting the considerably larger collection area provided by the array compared to a single antenna. Without suffering from optical losses, the optical probe beam can travel along a large circuit of TFLN waveguides and interact with the enhanced THz near-field generated at the gap of several antennas.

Our THz-antenna-driven quasi-phase-matching mechanism ensures that all the collected fields contribute constructively at a selected THz frequency, thus effectively suppressing out-of-band sensitivity. This design is robust since the large and distributed detection area provides flexibility and high sensitivity under diverse types of THz beam illumination. Compared to other phase-matching mechanisms, our work avoids complex periodic poling techniques^63,64^ by quasi-phase-matching two electromagnetic waves of extremely distant spectral ranges, namely the THz and optical domains, via the lithographic patterning of field-enhancing THz antennas. Similarly to conventional quasi-phase matching, the periodicity of the array (analog to the poling period) and the number of antennas (analog to the number of the poled regions) determine the center frequency and the detection bandwidth of the detector, respectively. With our approach, we demonstrated a relative bandwidth as narrow as $BW=\frac{FWHM}{{f}_{PM}}=8.2 \%$ that is highly relevant for applications sensitive to out-of-band channel jamming attacks^14^.

Furthermore, we showed that the detector can operate as a THz beam profiler, encoding the in-plane THz field into the time coordinate. This is enabled by the nearly single-cycle response of individual antennas, off-resonance to the phase-matching frequency of the device. This scheme could already pair with a feedback loop to open up possibilities for optimizing the illumination of the detector, similar to quadrant detectors, a missing component in the THz frequency range. While our proposal does not yet provide full two-dimensional and real-time THz imaging capabilities compared to state-of-the-art bolometric^65–67^ or field-effect^68–70^ cameras, the sensitivity of our design to low THz field strengths, its linearity, and high dynamic range, and our proposed read-out methods are anticipated to sparkle future work in building more complex architectures such as a photonics-based THz camera by combining several devices in parallel with synchronous read-out.

All the functionalities shown here represent a significant step towards plug-and-play and deployable THz field-resolved detectors that can be adopted in practical scenarios. By further engineering both the optical waveguides and the THz antennas, control over the device spectral response can be achieved to enable simultaneous operation on multiple THz bands. Alternatively, the single-period array may be replaced by a collection of frequency-chirped antennas, properly spaced, to achieve broadband detection. Furthermore, the entire array could be designed to be sensitive to THz radiation propagating parallel to its axis, rather than perpendicularly (i.e. an end-fire array type^71,72^), paving the way for detection of THz waves locally generated on the same chip towards spectroscopy applications^46^. Our detector requires relatively low optical energies of around 10 pJ and thus can be readily integrated with modern chip-scale femtosecond sources^73,74^. By integrating well-established telecommunication photodiodes on the same device can enable a chip-scale THz detector, paving the way toward the realization of fully integrated THz spectroscopy systems, time-of-flight measurements, and THz communications, all in a single and portable miniaturized device.

## Methods

### Design and fabrication of the MZM devices

A graphical representation of the device is presented in Fig. 1c. The optical circuit is fabricated starting from a 600-nm-thick film of X-cut lithium niobate (LN), bonded onto a 500-μm-thick high-resistivity silicon substrate stacked with a 2-μm-thick buffer layer of thermally-grown silicon dioxide (SiO2). A rib waveguide with a 1.5-μm-wide core is realized by etching 300 nm of the LN film, with a sidewall angle of *θ* = 63^∘^^51^. The waveguide then splits into two arms through a 50/50 directional coupler so as to form a Mach Zehnder interferometer configuration (MZI). A series of gold bow-tie antennas is deposited across the two arms of the MZI. The two antenna arrays are displaced along their arms by a length dependent upon the operating phase-matching frequency of each device (see main text). The lateral distance between the arms of the MZI is 670 μm in all devices. The waveguides are separated from the 15/285-nm Ti/Au electrode contacts by a distance of 0.9-μm thick, on each side, thus forming a total antenna gap of G = 3.3 μm. This value has been determined in such a way as to relieve plasmonic losses due to the leaking of the optical mode outside the waveguide core^46^. The antenna gap is filled with an 800-nm-thick Inductively Coupled Plasma Chemical Vapor Deposition SiO2 layer, which acts as a cladding material for the LN waveguide (see Supplementary Fig. 2). Finally, the optical circuit features a pair of grating couplers realized at each end of the MZI to couple in/out the optical probe beam from/to free space.

### THz time-domain-spectroscopy setup

The experimental setup is fed by a femtosecond laser oscillator (C-Fiber 780, Menlo Systems), free-space coupled and providing two beamlines, one at 1560 nm (first harmonic) and another one at 780 nm (second harmonic) wavelength. The 780 nm line excites an LT-GaAs PCA antenna, emitting THz pulses while biased by a 12-V-square wave voltage, oscillating at 5 kHz. A series of four 90^∘^, off-axis parabolic mirrors collect, collimate, and refocus the THz beam onto the final detector under nearly diffraction limit conditions. The 1560 nm line acts as the probe beam. Because of the operation with the integrated devices, for the sake of a fair comparison between the detection methods, the probe beam is fiber-coupled to a standard 2-m-long single-mode fiber (SMF28). When operating the free-space EOS to acquire the reference THz pulse, the probe beam is again coupled in free space and sent to a <110> 3-mm-thick GaAs crystal, which provides a superior collinear phase-matching condition at the 1560 nm wavelength, compared to, e.g., a ZnTe crystal (see Fig. 1). After interaction with the THz wave, the probe beam is acquired by an amplified, balanced photodiode pair (Nirvana, Newfocus), connected to a lock-in amplifier (UHFLI, Zurich Instrument). The latter is synchronized to the PCA bias modulation frequency. In the on-chip EOS configuration, the GaAs crystal is replaced by the MZI devices, while the 2-m-long fiber carrying the probe beam is terminated on an etched fiber tip, which illuminates the grating coupler. A second etched fiber tip collects the probe beam out-coupled from the second grating placed at the opposite side of the optical circuit sends it to a single channel of the balanced photodetector, which now operates in the single-ended configuration. Lock-in acquisition is carried out at the bias modulation frequency, as for the free-space EOS case. The integrated devices were fed with an estimated 4-mW-probe power, coupled through gratings and directly traveling along the waveguide. The THz waveforms were recorded by acquiring the readout signal generated by the photodiode while scanning the temporal delay between the probe and THz pulses.

## Supplementary information

Supplementary information of our article for online publication

## Data Availability

The data generated in this study will be made publicly available in the Zenodo database under 10.5281/zenodo.16881003.

## Code availability

The code used to plot the data within this paper is available in the Zenodo database under 10.5281/zenodo.16881003.
