# Supplementary material for: Large-area photonic circuits for terahertz detection and beam profiling
Source: Light Sci Appl. 2026 Jan 1;15:9. doi: 10.1038/s41377-025-02089-1 (PMC12756233; doi:10.1038/s41377-025-02089-1)
Supplement: Supplementary file 1 — Supplementary information of our article for online publication [file 41377_2025_2089_MOESM1_ESM.pdf]

Supplementary information for

Large-area photonic circuits for terahertz detection and beam profiling

A. Tomasino, A Shams-Ansari, M. Lončar, I.-C. Benea-Chelmus

Contents

1 Supplementary Note 1: Device parameters **3**

2 Supplementary Note 2: Terahertz Time Domain Spectroscopy setups for experimental characterizations **3**

3 Supplementary Note 3: Spatio-temporal knife-edge technique for THz beam profiling **5**

4 Supplementary Note 4: Analytical modeling of the quasi-phase-matching mechanism occurring in arrays of THz antennas **8**

5 Supplementary Note 5: Temporal interpretation of the formation of the THz transients recorded via antenna arrays. **12**

6 Supplementary Note 6: Comparison between Free-Space Electro-Optic Sampling technique and the electro-optic detection in TFLN chips **15**

7 Supplementary Note 7: Dependence of the frequency response of the device on the angle of incidence of the input THz beam **16**

1 Supplementary Note 1: Device parameters

The fabrication process of our samples is explained in the methods section. The dimensions of the samples are listed in the Supplementary Table 1. For some antenna parameters, we give a certain range, since these values were changed for different investigations.

thickness of silicon substrate:	H_{Si}	500 µm
thickness of silicon oxide isolation layer:	$H_{\mathrm{SiO_2}}$	4.7 µm
thickness of lithium niobate slab layer:	H_{slab}	300 nm
thickness of silicon oxide cladding layer:	H_{clad}	1100 nm
gold thickness:	H_{Au}	300 nm
length of bow-tie arm:	L_{ant}	90 µm
inner bow-tie arm width:	w_{ant}	5 µm
outer bow-tie arm width:	W_{ant}	30 µm
gap length:	l_{gap}	60 µm
electrode gap width:	w_{bar}	2 µm
antenna gap width:	w_{gap}	3 µm
waveguide width:	w_{wg}	1.5 µm
waveguide height:	H_{wg}	600 nm
distance between two antennas (same arm):	D_1	266 µm
distance between two antennas (opposite arm):	D_2	133 µm
length of the antenna array:	L_{MZM}	2.6 mm
width of the MZM:	W_{MZM}	677 µm

Supplementary Table 1: List of antenna and waveguide design parameters.

2 Supplementary Note 2: Terahertz Time Domain Spectroscopy setups for experimental characterizations

A simplified sketch of the two-color time-domain spectroscopy setup is shown in Supplementary Fig. 4. The Menlo C-780 fiber laser provides a train of 60-fs-long optical pulses (with an extremely broad spectrum ranging from 1420 nm to 1600 nm) and a peak average power of 550 mW at a 100 MHz repetition rate. The laser also provides a second output at the second harmonics (780 nm) of the nominal fundamental wavelength (1560 nm). Since they both originate from the main beam, the timing of the two pulses at the two different colors can be easily retrieved. Therefore, we use the 780 nm beamline to pump an LT-GaAs photoconductive (PCA) antenna, which acts as a THz source. The average pump power used is 100 mW, loosely focused to cover the active area of the PCA. The antenna is biased with a bipolar square wave having a peak-to-peak amplitude of 12 V, on-off modulated at 5 kHz. The emitted THz pulsed beam is collected through a 2-inch-diameter off-axis parabolic mirror (OAM1), with a 2-inch focal length. The latter forms a THz beam with a mean diameter of around 1.2 cm. Such a THz beam is subsequently handled by a series of parabolic mirrors (OAM2-3-4), which expand the beam diameter by a factor of two before being tightly focusing it onto the final detector. On the detection side, the probe beam is coupled into a single-mode fiber (SMF28) and then coupled out again in air to carry out free-space electro-optic sampling (FS-EOS) in a 1-mm-long <110>

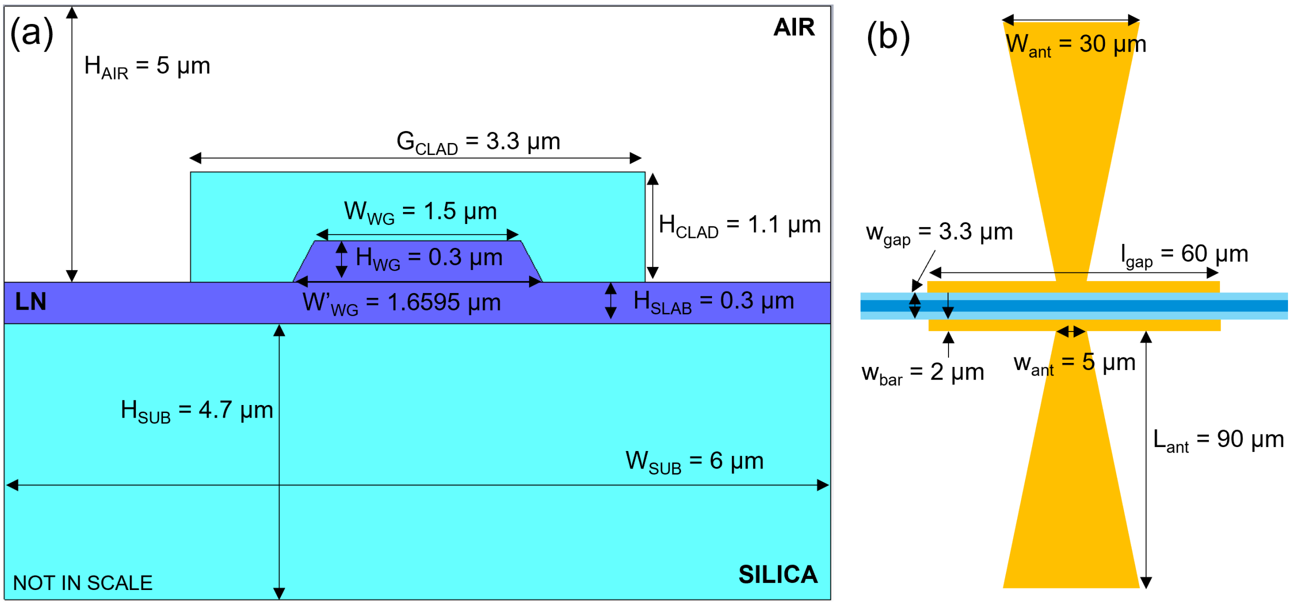

Supplementary Fig. 1: Schematics (a) of the waveguide geometry used to perform simulations in CST Microwave Studio and (b) of the top view of the THz antenna layout. All numbers are listed in the table 1.

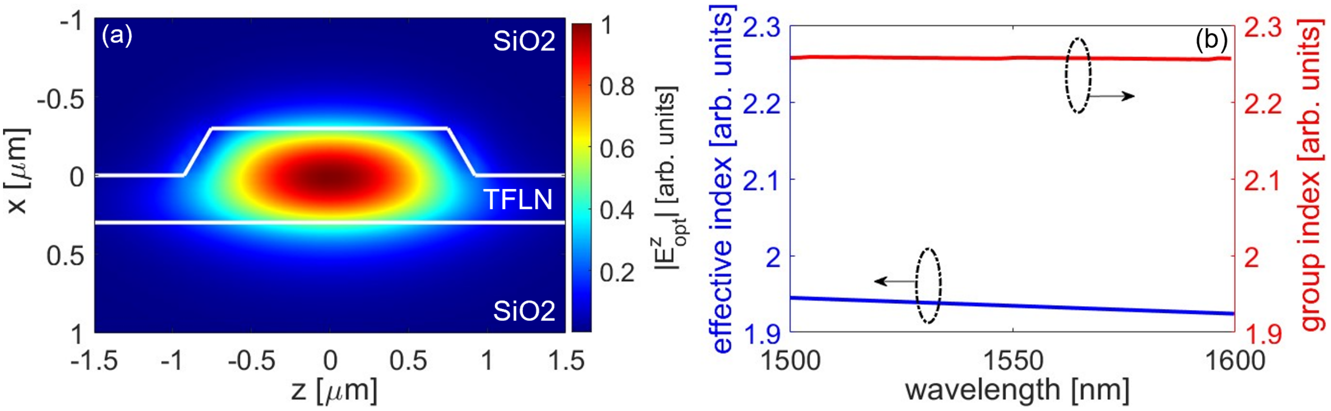

Supplementary Fig. 2: (a) Simulated electric field profile of the fundamental mode supported by the TFLN waveguide. The mode is polarized along the z-axis. (b) Effective refractive index (blue solid line) and group index (red solid line) of the fundamental mode as a function of the wavelength in the telecom range.

GaAs crystal (indicated as EOX in Fig. 4). The probe beam is focused using a 30-cm-lens and then re-collimated - after interacting with the THz pulse - by a 5-cm-lens, thus ensuring a complete illumination of the sensitive area of the balanced photodiode pair (BPD, Nirvana, Newfocus). Acquisition of the reference THz waveform is performed via lock-in detection (synchronized to the bias modulation frequency, 5 kHz) of the differential signal generated by the BPD while scanning the delay between the THz and the probe pulses. The latter is mechanically introduced by a delay stage placed on the optical pump path and controlled through a software application.

To carry out the on-chip electro-optic sampling, the probe beam path is modified as depicted in Supplementary Fig. 4. Specifically, we bypassed the second fiber coupler, and directly connected the SMF fiber to a free-standing "fiber-probe". The latter consists of a short bare fiber patch that terminates with a cleaved facet. This way, the light beam emitted from the fiber probe shines on top of the grating coupler realized on the chip. To do so, we used a piezoelectric module that enables extremely fine movements with a step size in the order of nanometers. A CCD camera images the chip, allowing the operator to pinpoint the position of the grating couplers, as well as keep track of the relative position between the MZI device and the focal point of the parabolic mirror (which, in turn, indicates the position of the THz beam spot).

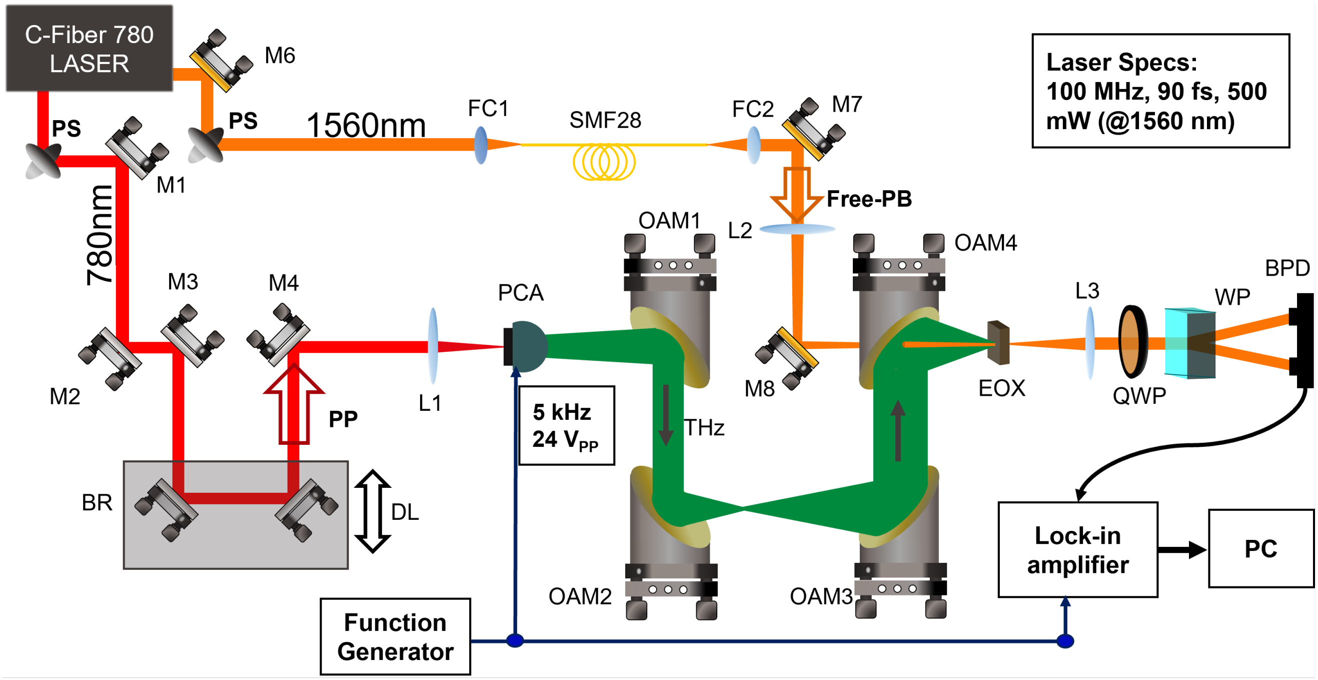

Supplementary Fig. 3: Two-color THz Time-Domain Spectroscopy set-up. An optical beam at a wavelength of 780 nm drives a photoconductive antenna (PCA) emitting a burst of THz pulses. The THz beam is handled by a series of 4 parabolic mirrors (OAM) and finally focused onto a bulk electro-optic crystal (EOX). The 1550-nm probe beam overlaps in time and space with the THz beam into the EOX crystal to carry out free-space electro-optic sampling. The THz-induced polarization modulation of the probe beam is revealed by using a quarter waveplate (QWP) and a Wollaston prism (WP), which split and send the two polarization components to a balanced photodiode pair (BPD). A function generation feeds the PCA with a square wave bias voltage, also providing the reference signal for the lock-in amplifier that acquires the readout signal from the BPD.

The out-coupled probe beam is collected through the use of a second fiber probe, controlled by a second piezoelectric stage, and operated reciprocally with respect to the previous one. The light is then sent to a single photodiode. Note that no differential technique could be implemented in this configuration as the output of the interferometer is single-ended. Acquisition of the THz waveform is performed in the same manner as the free-space case.

3 Supplementary Note 3: Spatio-temporal knife-edge technique for THz beam profiling

In this section, we explain how we characterized the THz beam in terms of the radial dimension associated with each frequency component. To do so, we carried out a modified version of the well-known knife-edge technique for conventional optical beams [1]. We recall that a traditional knife-edge technique [2] assumes that the emitted beam exhibits an electric field profile with separable temporal and spatial distributions. This is usually the case for a beam either emitted by a large-area source (i.e., larger than the wavelength squared) or collimated with very long Rayleigh ranges. In this case, the intensity profile can be decomposed into the product of two functions solely dependent upon one single transverse coordinate. In formulae, if the beam propagates along the z direction and its intensity profile $I(x, y)$ is distributed on the xy-plane, the separability condition allows to write: $I(x, y) = I_x(x)I_y(y)$. When a blade is inserted in the xy-plane and cuts the beam, for instance, along the x-axis up to the coordinate x_0, the power $P(x_0)$ unblocked by the blade and reaching an optical detector can be written as:

$$P(x_0) = \int_{-\infty}^{x_0} I_x(x)dx \int_{-\infty}^{+\infty} I_y(y)dy \tag{1}$$

By varying x_0 through the translation of the blade across the entire beam size, the function $P(x_0)$ can be reconstructed (with x_0 being now a continuum variable). The intensity profile can be then retrieved by differentiation of Eq. 1.

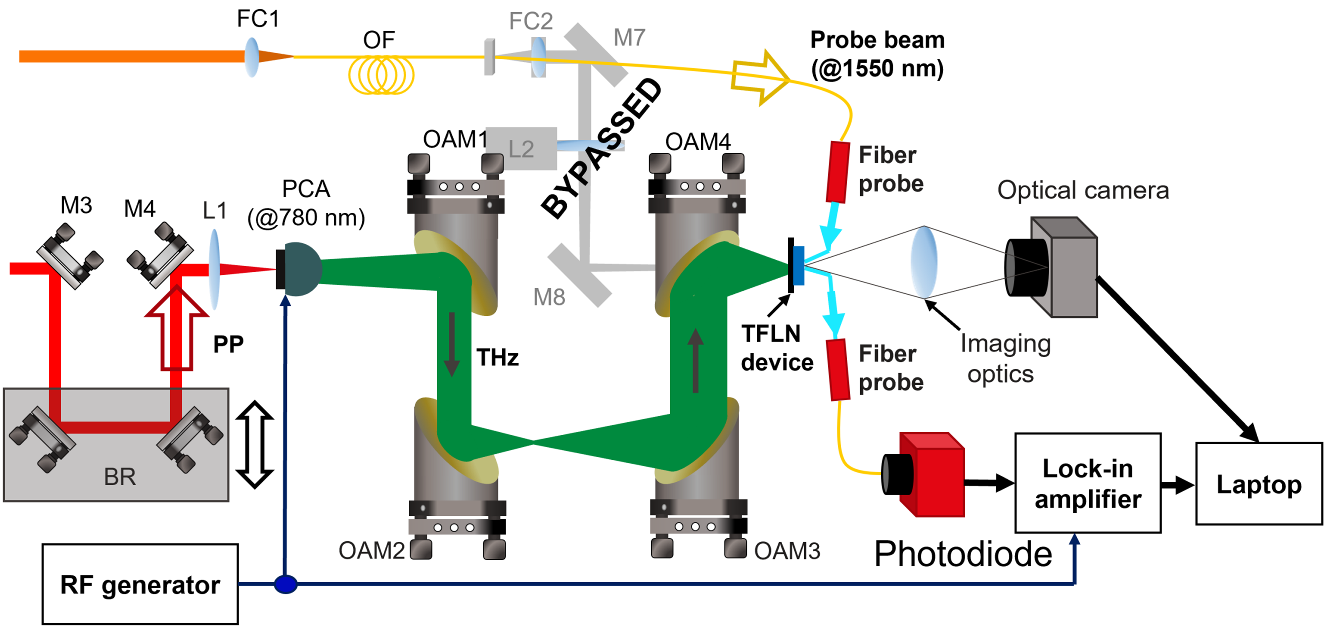

Supplementary Fig. 4: Modified two-color THz Time-Domain Spectroscopy set-up for on-chip electro-optic sampling. The generation path is unchanged. On the detection side, the probe beam is coupled in and out of the chip through the fiber probes placed on top of grating couplers. The chip is placed on the THz focus and imaged via a CCD camera to allow alignment of the fiber probes. The out-coupled probe beam is sent to a single-ended photodiode for acquisition.

Using the same procedure along the y-axis, it is possible to reconstruct $P(y)$ and the associated intensity profile, thus providing the entire characterization of the beam dimensions. The application of this technique to THz beams is generally not of an immediate implementation. Indeed, owing to the sub-wavelength geometries often adopted to generate THz beams, the standard knife-edge technique fails to reproduce the exact spatial profile, since the temporal and spatial coordinates couples along the propagation. However, in this work, the THz beam is emitted by a large aperture PCA provided with a hyper-hemispherical silicon lens that forms an emerging beam with a size comparable to the lens radius (around 0.5 cm). In addition, the series of parabolic mirrors in our setup forms a THz beam with a diameter of a few centimeters, thus making the use of the knife-edge technique possible. Furthermore, the access to the electric field waveforms through time-domain measurements (i.e., to both its amplitude and phase) can be exploited to perform a superior implementation compared to the optical case. More in detail, in the field-resolved system depicted in Fig.4 of the main manuscript, where the transverse plane is zy, when the blade cuts a spatio-temporal THz beam, the uncut section of the beam becomes a new source of radiation with a resultant electric field $E^{res}_{THz}(z_0, t)$ proportional to the incident electric field $E^{in}_{THz}(z, t)$ as:

$$E^{res}_{THz}(z_0, t) \propto \int_{z_0}^{\infty} E^{in}_{THz}(z, t)dx \propto \frac{E_0}{2}\text{erfc}(\frac{z - z_0}{R_z}) \tag{2}$$

where z_0 is the blade position, while we are assuming that the input THz beam resembles a Gaussian spatial profile ($E^{in}_{THz}(z) \propto E_0 \exp[(-z/R_z)^2]$), with R_z being the radius of the collimated beam that we want to estimates.

In Eq. 2, 'erfc' stands for the complementary error function. The same considerations apply to the y-direction, leading to the estimation of R_y. Therefore, by performing several acquisitions as a function of the blade position z_0, the transverse profile of the collimated THz beam can be retrieved by differentiating Eq. 2 with respect to the z-coordinate. In practice, we utilized the FS-EOS configuration to record a series of the THz waveforms for different blade positions inserted in the THz beam path right in front of OAM2, with focal length f_{OAM}. This way, the uncut section of the THz beam will be straightforwardly captured and focused by the parabolic mirror into the detection crystal. Supplementary Fig. 5a shows the waveforms recorded as a function of blade position, whereas Fig. 5b

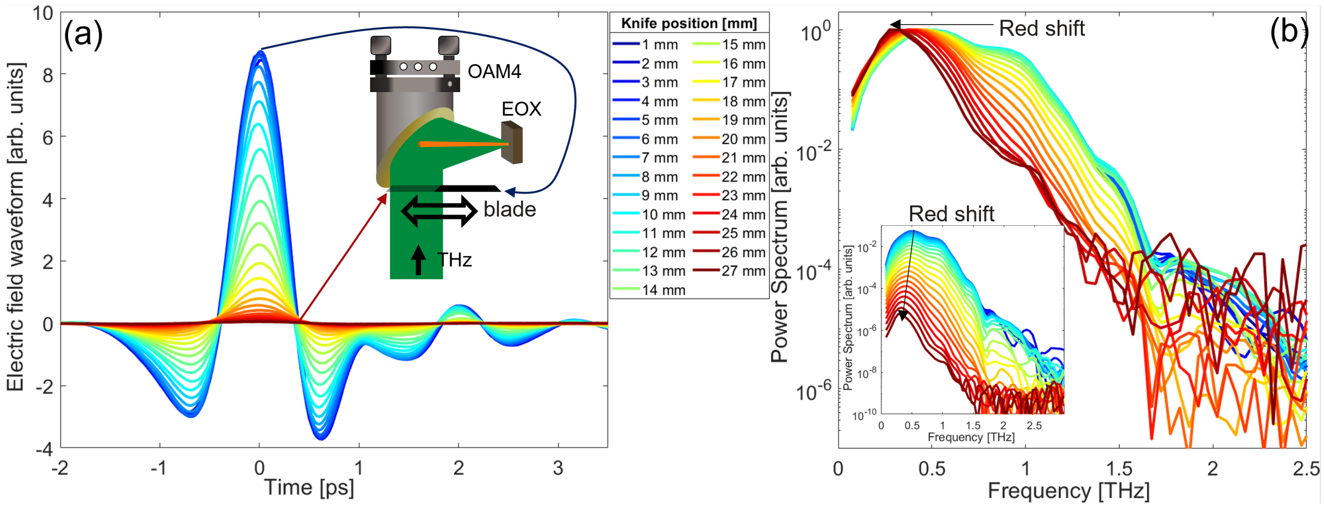

Supplementary Fig. 5: Time-Domain Spectroscopy of a transversely cut THz pulsed beam. (a) THz waveforms retrieved as a function of the relative position of the blade inside the THz beam. (b) FFT spectra of the waveforms in (a). Curves are normalized with respect to their maximum. The inset shows the non-normalized spectra. As the blade cuts deeper into the beam, the peak of the reconstructed spectra moves towards lower frequencies.

represents the FFT-calculated spectra corresponding to the waveforms in panel (a). Larger numbers for the blade position correspond to a deeper insertion into the THz beam path. Since a THz beam is quite broadband, it could be regarded as a superposition of a virtually infinite number of beams at different frequency components. As lower frequencies diffract out in larger radiation patterns compared to higher frequencies, it is plausible to assume that the periphery of the THz beam is more low-frequency rich compared to its central part. Conversely, the energy associated with low-frequency components is spread across a much larger area. Because of this, as the blade moves deeper into the THz beam, not only does the amplitude of the acquired THz transient diminish (see Supplementary Fig. 5a), but also their waveforms and consequently their spectra experience a significant reshaping. In particular, we observe a noticeable red-shift of the calculated peak frequency (Supplementary Fig. 5b), which has to be ascribed to the fact that the blade is more efficient in filtering high-frequency components for deeper cuts, because of the reduced spatial extension compared to those at lower frequencies. By taking the peak values of each temporal amplitude and plotting them as a function of the blade position along z, we constructed the curve displayed in Supplementary Fig. 6a. The data points are then fitted with the first integral of a Gaussian function, as described in Eq. 2. The good fit between the model and data points proves the good quality of the formed THz beam. This fit returns a mean beam radius of $R_z = 6.16$ mm. The same procedure returns $R_y = 5.84$ mm. Here, the term 'mean' is used to stress the fact that it accounts for an average value across the whole THz spectrum. To retrieve more specific values, we take advantage of the time-resolved knife-edge technique. Indeed, similarly to the time data, we can build a graph similar to that in Supplementary Fig. 6a, yet in a dense range of frequency components, as reported in Supplementary Fig. 6b. We repeated the same procedure by cutting the beam along the y-axis and obtaining the plots in Supplementary Fig. 6c-d. By fitting the spectral curves in Supplementary Fig. 6b-d with different 'erfc' functions, we can reconstruct a trend of the beam radius as a function of the THz frequency components, which are reported in Supplementary Fig. 7a and b, for the z-axis and y-axis, respectively. It results that the THz beam has a frequency-dependent beam radius (R_z^f) slowly decreasing with the frequency, ranging approximately between $R_z^f = 4.5$ mm - 8 mm, in the spectral window between 0.2 and 1.5 THz. Finally, by using the laws of Fraunhofer diffraction, we can reconstruct the profile of the THz electric field at the focal point of the parabolic mirror, by taking the spatial Fourier transform of the

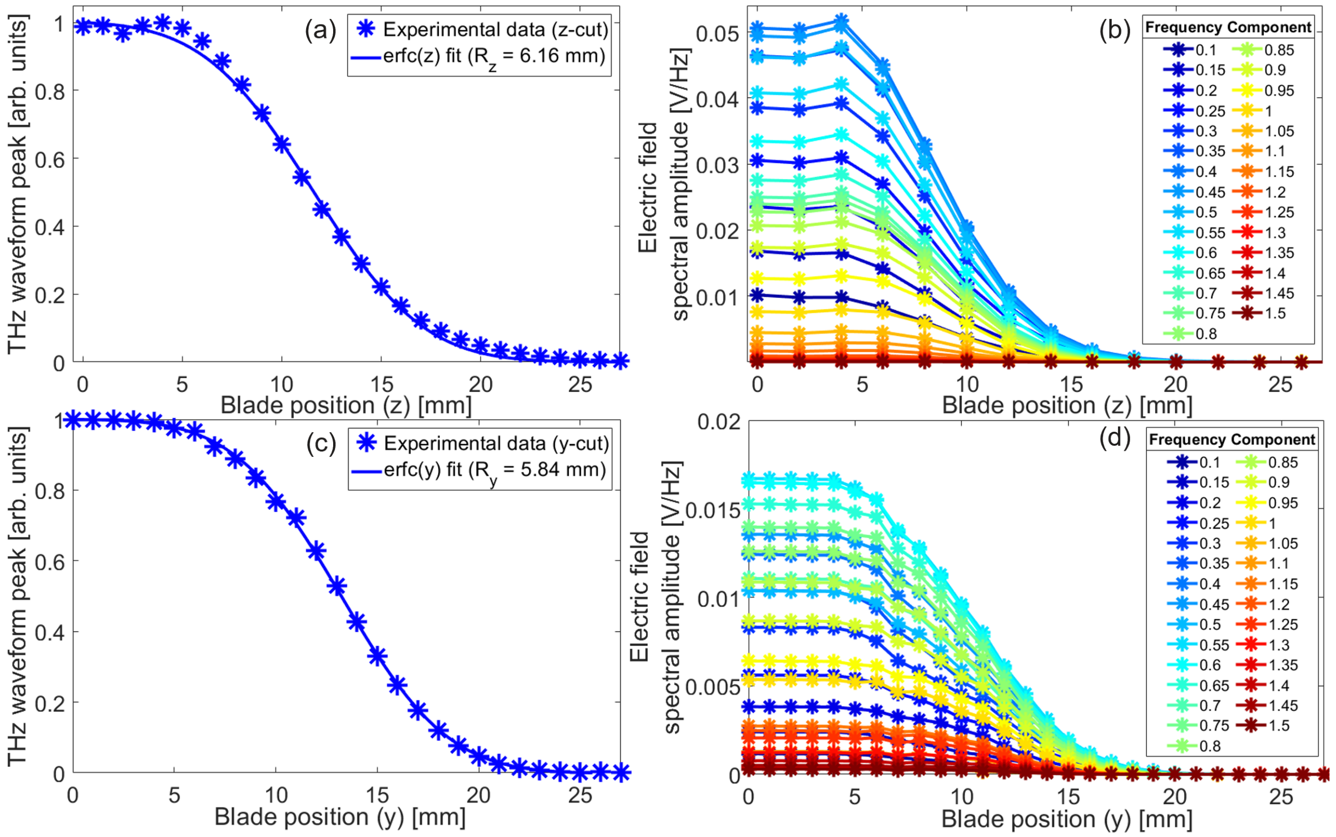

Supplementary Fig. 6: Knife-edge characterization of the THz beam. Amplitude peak of the THz waveform recorded as a function of the blade position (blue dots) fitted with the theoretical error function ('erfc') characterizing ideal Gaussian beams (blue solid line) for the case of horizontal cut along the z-axis (a) and vertical cut along the y-axis (c). (b) Peak value trends of the spectral amplitude for selected frequency components as a function of the blade position, for the (b) horizontal and (d) vertical cut.

collimated beam profile (i.e., the spatial derivative of Eq. 2):

$$E_{THz}^{meas}(z',t) = \int_{-\infty}^{+\infty} E_{THz}^{in}(z,t)e^{-ik_z z}dz \propto \int_{-\infty}^{+\infty} E_0 e^{(-z/R_z)^2} e^{-ik_z z}dz \propto \exp\left[\left(-\frac{\pi R_z z'}{f_{OAM}\lambda}\right)^2\right] \tag{3}$$

where $k_z = kz'/f_{OAM}$, being $k = 2\pi/\lambda$ the THz wavevector, and z' the conjugate spatial coordinate of z in the focal plane of the parabolic mirror (reciprocal plane). From Eq. 3, we derive that the mean waist size w_{THz}^{z} at the final focal plane can be calculated as: $w_{THz}^{z} = \frac{\lambda f_{OAM}}{\pi R_z} = 660\mu m$. Once again, a similar equation to that in Eq. 3 can be utilized for each frequency component, which allows to reconstruct the trend of the waist size as a function of the frequency. The latter is reported in Supplementary Fig. 7a, showing a THz waist ranging between 3.2 - 0.375 mm in the 0.2 -1.5 THz range along the z-axis. Supplementary Fig. 7b shows the frequency dependent waist size calculated for the for the y-axis, showing slightly larger values than the z-axis case, due to the fact that the collimated beam radius are in opposite relationship (i.e., $R_y < R_z$). The mean waist size along the y direction is $w_{THz}^{y} = \frac{\lambda f_{OAM}}{\pi R_y} = 700\mu m$. These two values are reported in Fig. 3h of the main manuscript.

4 Supplementary Note 4: Analytical modeling of the quasi-phase-matching mechanism occurring in arrays of THz antennas

In this section, we derive the spectral response of the TFLN device in terms of its intensity transmission function T_{MZI} reported in Eq. 1 of the main manuscript. We start the digression by considering the scheme in Supplementary Fig. 8a. It depicts a conventional MZI geometry, where each arm imparts a different phase retardation to each of the two optical beams guided through two distinct components, represented as the red (up) and green (low) blocks.

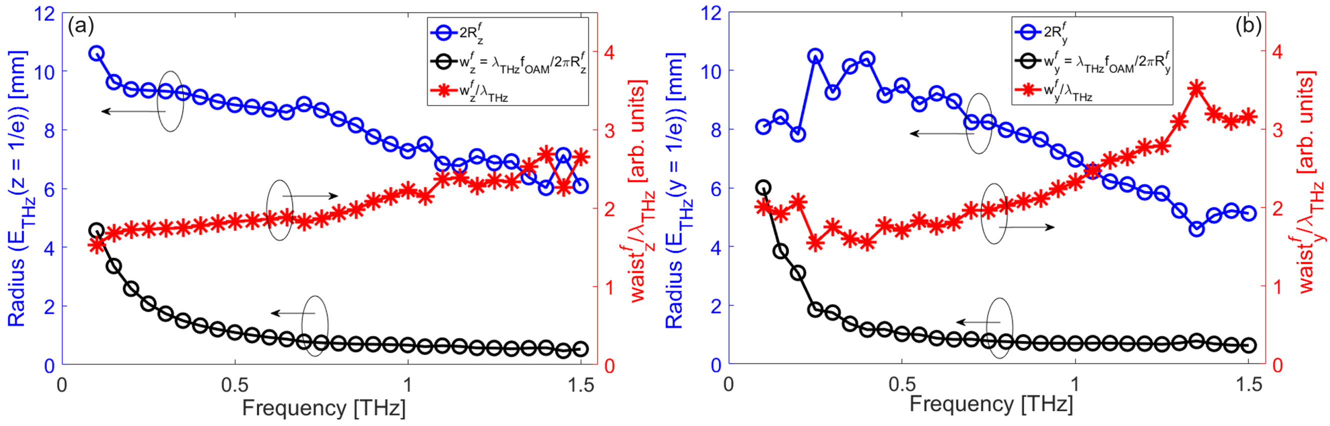

Supplementary Fig. 7: Reconstruction of the THz beam radii and waist sizes as a function of the frequency. (a) Calculated collimated beam size (blue dotted line) and waist size (black dotted line) as a function of the frequency. The red stars curve represents the Rayleigh ratio indicating that the THz source operates above the diffraction limit.

Here, we assume that the input optical beam has an electric field E_0^{in}, which splits into two components with identical initial amplitude $E_0^{in}/\sqrt{2}$ and phase (here, conveniently fixed at zero for simplicity). After propagating along each arm, the THz electric field established in the antenna arrays will modulate the two optical beams as:

$$E^U = \frac{E_0^{in}}{\sqrt{2}} e^{i(\phi_{THz}^U + \phi_Q)} \tag{4}$$

$$E^D = \frac{E_0^{in}}{\sqrt{2}} e^{i\phi_{THz}^D} \tag{5}$$

where ϕ_{THz}^U and ϕ_{THz}^D are the phase retardation experienced by the upper and lower beam, respectively. Here, we are still treating a generic case where the two modulations might differ between arms. The term ϕ_Q accounts for the built-in dephasing between the two arms, intentionally introduced to operate the MZI device at its quadrature point (i.e., with its output intensity equal to half of that of the input). At the MZI output, the superposition of the two optical beams will give rise to:

$$E_{out} = \frac{E^U + E^D}{\sqrt{2}} = \frac{E_0^{in}}{2}\left[e^{i(\phi_{THz}^U + \phi_Q)} + e^{i\phi_{THz}^D}\right] = \frac{E_0^{in}}{2} e^{i\frac{\phi_{THz}^U + \phi_Q + \phi_{THz}^D}{2}} \left[e^{i\frac{\phi_{THz}^U + \phi_Q - \phi_{THz}^D}{2}} + e^{-i\frac{\phi_{THz}^U + \phi_Q - \phi_{THz}^D}{2}}\right] \tag{6}$$

The quadrature point corresponds to the condition $\phi_Q = \pi/2$, thus, after applying further simplifications, we achieve:

$$E_{out} = E_0^{in} e^{\frac{i\pi}{4}} e^{i\frac{\phi_{THz}^U + \phi_{THz}^D}{2}} \cos\left[\frac{\phi_{THz}^U - \phi_{THz}^D}{2} + \frac{\pi}{4}\right] \tag{7}$$

Since the photodetector is sensitive to the intensity of the probe electric field in Eq. 7, we can estimate it as:

$$I_{out} \propto |E_{out}|^2 = |E_0^{in}|^2 \cos^2\left[\frac{\phi_{THz}^U - \phi_{THz}^D}{2} + \frac{\pi}{4}\right] = \frac{|E_0^{in}|^2}{2}\left[1 + \cos\left(\phi_{THz}^U - \phi_{THz}^D + \frac{\pi}{2}\right)\right] \tag{8}$$

Now, by defining $\Delta\phi_{THz} = \phi_{THz}^U - \phi_{THz}^D$ and using $I_{in} \propto |E_0^{in}|^2$, we can finally write:

$$I_{out} = \frac{I_0^{in}}{2}\left[1 + \sin\Delta\phi_{THz}\right] \tag{9}$$

By taking the difference between the readout intensity measured with and without THz illumination, we can write the THz-induced intensity modulation as:

$$\Delta I_{out} = I_{out}\left(E_{THz} \neq 0\right) - I_{out}\left(E_{THz} = 0\right) = \frac{I_0^{in}}{2}\sin\Delta\phi_{THz} \approx \frac{I_0^{in}}{2}\Delta\phi_{THz} \tag{10}$$

where we have used the fact that $\Delta\phi \ll 1$ under weak THz modulation. Finally, we can rearrange Eq. 10 as:

$$\frac{\Delta I_{out}}{I_{out}^Q} \approx \Delta\phi_{THz} \propto E_{THz} \tag{11}$$

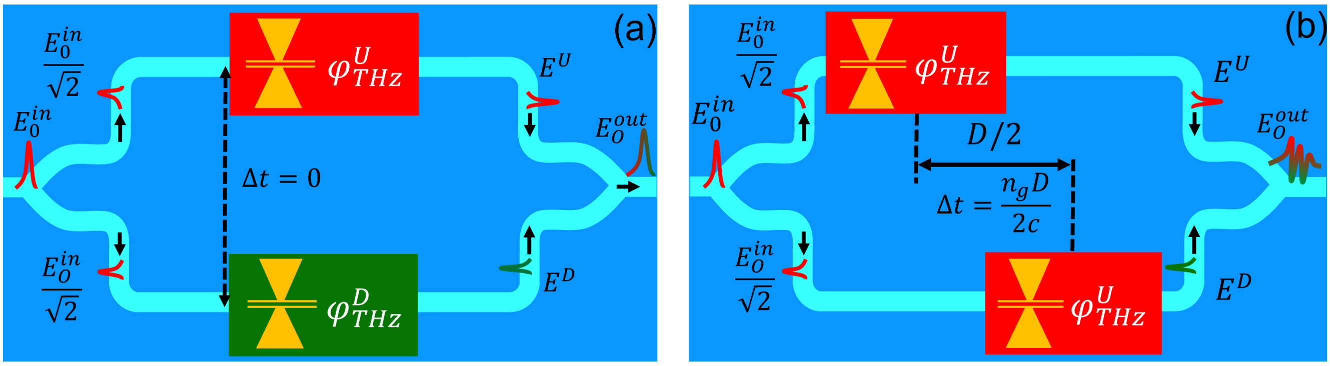

Supplementary Fig. 8: Electro-optic modulation in a Mach Zehnder interferometer hosting arrays of THz antennas. The input optical beam E_0^{in} is split into two sub-beams with identical initial amplitude and phase. (a) In the symmetric case, the two sub-beams undergo the same phase modulation (ϕ^U_{THz} and ϕ^D_{THz} for the upper and lower arm, respectively) as each beam encounter the array on (red and green boxes) at the same time instant. The two sub-beam recombine generating an output beam that carries twice the phase modulation of the single arm case. (b) In the displaced case, the same array is translated along the arm by a length D/2, so that the lower sub-beam has to travel for an extra time delay Δt before interacting with the array. The unbalancing of the two different phase modulations leads to the amplitude modulation of the recombined beam at the output.

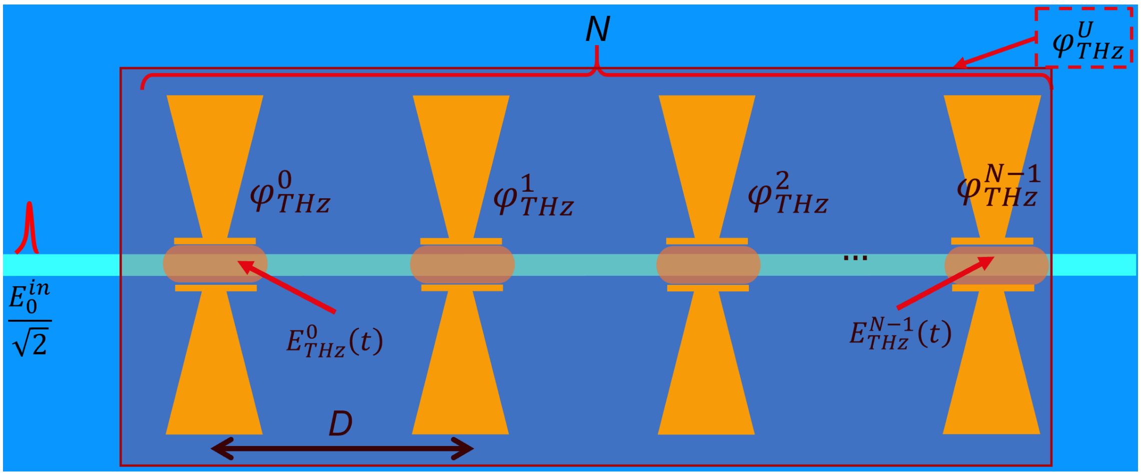

Supplementary Fig. 9: Building-up of the phase modulation in an array of THz antennas. The sub-probe propagates through an optical waveguide that crosses the gaps of an array of N antennas with a spatial period D (semi-transparent red box). At each antenna encounter, the probe beam experiences a phase modulation ϕ^n_{THz}, with$n = 0, ..., N-1$ imparted by the instantaneous THz electric field $E^n_{THz}(t)$, with$n = 0, ..., N-1$ established at each gap. The summation over all contribution construct the total ϕ^U_{THz} due to a single arm of the interferometer.

where $I^Q_{out} = I^{in}_0/2$ is the intensity output of the MZI at its quadrature point. Equation 11 expresses the linear dependence of the variation of the relative probe intensity upon the THz electric field, thus implying a phase-sensitive reconstruction of the THz wave. Let us now consider the case that the phase retardation is imparted to the two probe beams at a different time instant, as indicated in Supplementary Fig. 8b. In particular, the two blocks are spaced by a distance $D/2$, which corresponds to a time delay of $\frac{T}{2} = \frac{n_g D}{2c}$, where n_g is the group refractive index, and c the speed of light in vacuum. Let us also assume that the two blocks apply the same phase modulation when taken singularly. Then, the lower phase modulation will only differ from the upper arm by an extra time delay, namely:

$$\phi^D_{THz}(t) = \phi^U_{THz}\left(t + \frac{T}{2}\right) \tag{12}$$

that can be expressed in the frequency domain as:

$$\phi^D_{THz}(\omega_{THz}) = \phi^U_{THz}(\omega_{THz})e^{i\frac{\omega_{THz}T}{2}} \tag{13}$$

Therefore, the total phase modulation imparted at the output of the interferometer can be written in the frequency

domain as:

$$\Delta\phi(\omega_{THz}) = \phi^U_{THz}(\omega_{THz}) - \phi^U_{THz}(\omega_{THz})e^{-i\frac{\omega_{THz}T}{2}} = \phi^U_{THz}(\omega_{THz})\left(1 - e^{i\frac{\omega_{THz}T}{2}}\right) = \\ = \phi^U_{THz}(\omega_{THz})\left[-2ie^{i\frac{\omega_{THz}T}{4}}\sin\frac{\omega_{THz}T}{4}\right] \tag{14}$$

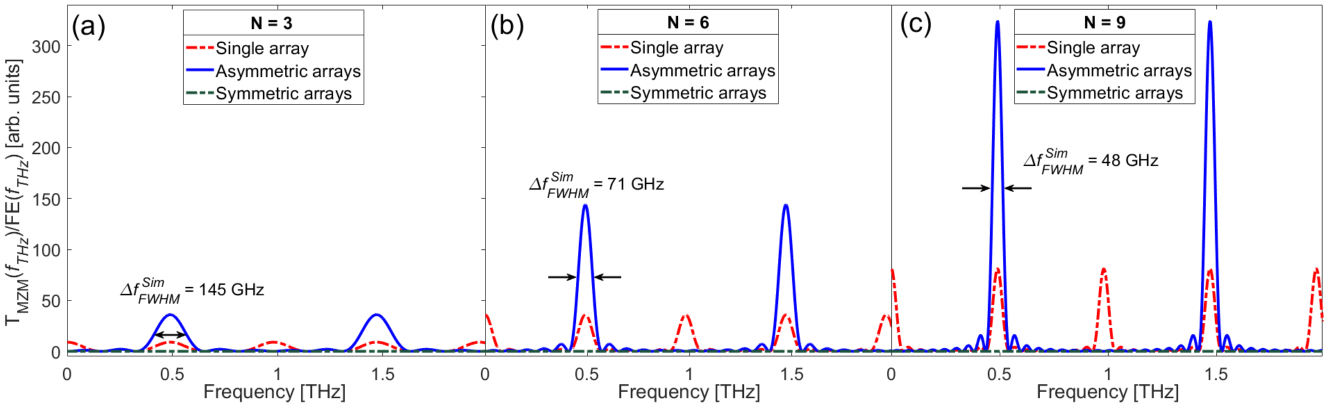

Supplementary Fig. 10: Spectral transfer function (T_{MZI}) of the MZI devices as a function of the number of antennas red dotted line), double symmetric (green dotted lines) and displaced array (blue solid line) for (c) $N = 3$, (d) $N = 6$, (e) $N = 9$ antennas, as shown in (a) Amplitude peak of the THz waveform as a function of the blade position (blue dots) fitted with the theoretical error function characterizing ideal Gaussian beams (blue solid line). (b) Trend of peak values of selected frequency components as a function of the blade position. (c) Calculated collimated beam size (blue dotted line) and waist size (black dotted line) as a function of the frequency. The red stars curve represents the Rayleigh ratio indicating that the THz source operates above the diffraction limit.

Equation 14 allows for the computation of the phase retardation due to both arms having the same type of antenna array, at any value of the delay T/2. We note that for $T/2 = 0$ (corresponding to the configuration in Supplementary Fig. 8a), the phase imbalance between the two arms is zero ($\Delta\phi(\omega_{THz}) = 0$) at any temporal instant, thus resulting in an identically null intensity modulation of the probe beam, as correctly predicted by Eq. 10. At this point, the only quantity left to be computed is the phase modulation due to a single antenna array. To this end, Supplementary Fig. 9 represents the upper sub-probe beam traveling consecutively through the gaps of a N-antenna array, separated by a distance equal to D from each other. The probe beam crosses each antenna at time instants spaced by the quantity $\Delta t = \frac{n_g D}{c} = T$, so that the delay accumulated increases with steps of $t_n = t_0 + nT$, with t_0 being the time at which the probe encounters the first antenna and $n = 0, 1, ..., N-1$ is an integer. As shown above, we stress that the delay T is purposely chosen as the double of the delay mutually separating the two opposite arms (i.e., $T/2$). In the time domain, the total phase retardation accumulated by the probe beam at the end of the array is then equal to a summation over all individual contributions:

$$\phi^U_{THz}(t) = \phi_{THz}(t_0) + \phi_{THz}(t_1) + ... + \phi_{THz}(t_{N-1}) = \sum_{n=0}^{N-1}\phi_{THz}(t_0 + nT) \tag{15}$$

Moving to the frequency domain, we obtain:

$$\phi^U_{THz}(\omega_{THz}) = \sum_{n=0}^{N-1}\phi^0_{THz}(\omega_{THz})e^{in\omega_{THz}T} \tag{16}$$

where $\phi^0_{THz}(\omega_{THz})$ is the Fourier Transform of $\phi_{THz}(t_0)$. We recognize in Eq. 16 the definition of array factor typical of a phased array [3]. The sum in Eq. 16 is simply a geometric series of ratio $e^{i\omega_{THz}T}$ and can be easily calculated as:

$$\phi^U_{THz}(\omega_{THz}) = \phi^0_{THz}(\omega_{THz})\frac{e^{i\omega_{THz}NT} - 1}{e^{in\omega_{THz}T} - 1} = \phi^0_{THz}(\omega_{THz})\frac{\sin\frac{\omega_{THz}NT}{2}}{\sin\frac{\omega_{THz}T}{2}}e^{i\frac{\omega_{THz}T(N-1)}{2}} \tag{17}$$

By merging the results of Eq. 17 and Eq. 14, we can write the total phase retardation imparted by interferometer to the output probe beam under THz illumination as:

$$\Delta\phi(\omega_{THz}) = -2i\phi^0_{THz}(\omega_{THz})e^{-i\frac{\omega_{THz}T}{4}}\frac{\sin\frac{\omega_{THz}NT}{2}}{\sin\frac{\omega_{THz}T}{2}}\sin\frac{\omega_{THz}T}{4}e^{i\frac{\omega_{THz}T(N-1)}{2}} \tag{18}$$

The information about the THz electric field is included in the term $\phi^0_{THz} \propto E^0_{THz}$, which also accounts for the spectral response of each antenna through its field enhancement factor $FE(\omega_{THz}) = E^0_{THz}/E^{in}_{THz}$, where E^{in}_{THz} is the impinging THz electric field from the free-space. Therefore, we can finally define the transfer function of the MZI with double displaced arrays, normalized to the THz electric field enhanced in each antenna as (Eq. 2 of the main manuscript):

$$\frac{\Delta\phi(\omega_{THz})}{FE(\omega_{THz})} = T_{MZI}(\omega_{THz}) \propto -2ie^{-i\frac{\omega_{THz}T}{4}}\frac{\sin\frac{\omega_{THz}NT}{2}}{\sin\frac{\omega_{THz}T}{2}}\sin\frac{\omega_{THz}T}{4}e^{i\frac{\omega_{THz}T(N-1)}{2}} \tag{19}$$

Equation. 19 can be used to predict the spectral sensitivity of different MZI devices featuring arrays with increasing antennas. More in detail, we here compare the symmetric and asymmetric (i.e., displaced) double array configurations. Supplementary Fig. 10 shows the calculated spectral responses for $D_1 = 2D_2 = 266$ μm, corresponding to a $f_{PM} =$ 487 GHz, and for three different values of $N = 3, 6, 9$, respectively, as shown in Fig. 4 of the main text. As expected, the symmetric double array ($\Delta t_2 = 0$) exhibits no sensitivity over the entire band, regardless of the number of antennas, since $T_{MZI}(f, T/2 = \Delta t_2 = 0) = 0$. Indeed, in the latter case, the two arms operate identically, effectively acting as a single entity (phase modulator). Therefore, the THz electric field modulation remains encoded into the probe phase, which is not detectable with an optical photodetector. Conversely, the built-in asymmetry of the displaced double array makes the device sensitive to relatively narrow spectral regions around the main f_{PM} and its odd harmonics only. For comparison, the single array case (which also corresponds to an inherent asymmetry between the arms of the interferometer) is displayed in Supplementary Fig. 10. The latter reveals an amplitude response four times smaller (in power) than that of the asymmetric double array, yet with extended sensitivity to the even harmonics. This has to be expected since the $T/2$ displacement featuring the asymmetric configuration leads the even harmonics of the single array case to destructively interfere upon recombination. Finally, we note that in both cases, the linewidth around the frequency components at a multiple of f_{PM} scales inversely with the number of antennas $\Delta f^{Sim}_{FWHM} \propto \frac{1}{N^2}$. This implies that a certain trade-off between detected bandwidth and amplitude response can be achieved by choosing a proper number of antennas. We point out the excellent agreement between the calculated linewidth and that retrieved experimentally, as shown in Fig. 2g of the main manuscript.

5 Supplementary Note 5: Temporal interpretation of the formation of the THz transients recorded via antenna arrays.

The digression presented in the previous section provides an analytical description of the spectral response of the whole MZI device. However, owing to the transient nature of the reconstructed THz signal, the operation of the device (specifically, of the antenna arrays) can be effectively described in the time domain, by considering the interplay of THz near-field oscillations established in the gap of each antenna as the mutual delay between the incoming THz and probe pulses is being changed. We recall from Supplementary Fig. 4 that in our setup the probe path is kept fixed, while the mechanical delay line varies the length of the THz beam path. In particular, all measurements shown throughout the main text have been recorded by moving the delay line along its forward direction, thus shrinking the THz path. Therefore, longer time delays in the temporal axis correspond to the THz pulse that arrives earlier at the device. Let us now consider Supplementary Fig. 11a, depicting the probe beam about to cross an array of

three antennas. Let us also assume that the delay line is stopped at a position that leads the probe beam to firstly encounter the THz pulse at the most right antenna (Ant. 1, blue gap), right at the time instant when the THz near-field oscillation exhibits its peak (marked as $\Delta\tau = 0$). In this case, since all antennas resonate synchronously, in order for the probe beam to be temporally aligned with the emergence of the THz peak at the last antenna, it has to cross the previous two antennas without experiencing any phase modulation, as the latter were not excited by the incoming THz electric field yet.

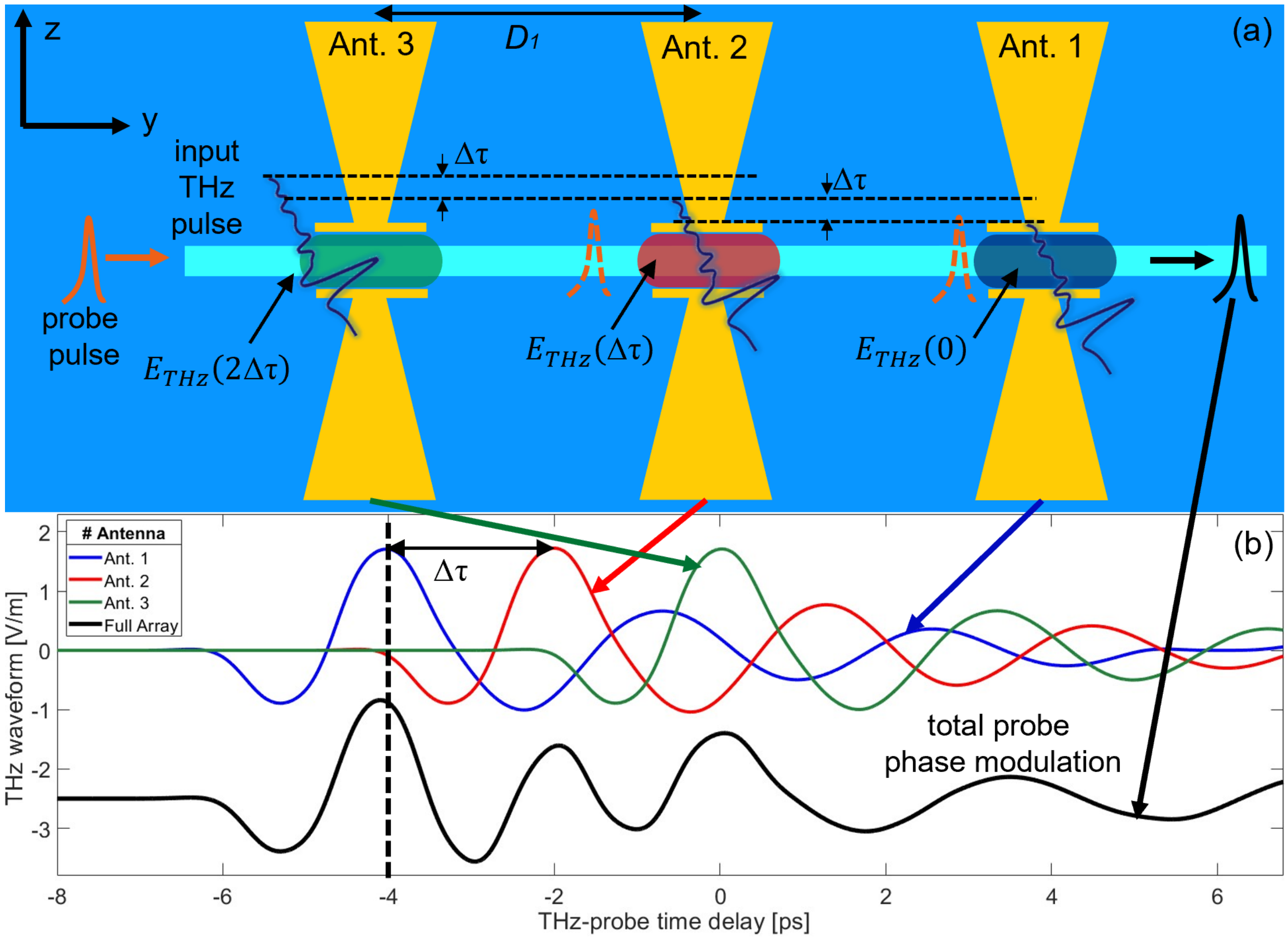

Supplementary Fig. 11: Time diagram of the THz-induced phase modulation of a probe beam imparted by an antenna array. (a) Sketch depicting an array of THz antenna illuminated simultaneously by an incident THz pulse. In these settings, the probe pulse path is fixed, while the THz pulse path is varied via an external delay line that scans the mutual delay between the probe and THz pulses. The specific time delay corresponding to the travel time the probe pulse takes to move between consecutive antennas is $\Delta\tau$. Note that the impinging THz pulse is drawn with a tilt angle only for the sake of clarity (the incidence axis is purely orthogonal to the array plane). (b) As the time delay increases, the THz pulse path shrinks, making the THz pulse arrive at the chip at ever earlier times. Thus, the probe pulse will effectively interact with the THz near-field of an ever-increasing number of antennas as it propagates towards the end of the array (green, red, and blue solid lines moving from earlier to later time delays). The most right antenna is considered as the reference for this time frame (vertical dashed line). The total phase modulation applied to the probe pulse (black solid line) is thus the temporally-spaced superposition of all antenna contributions. Note that while the blue, red and green curve represent actual THz electric field waveforms, the black curve is more properly a representation of the THz-induced probe phase modulation and it is vertically shifted for clarity only.

Since each antenna is temporally separated from its neighbors by a time interval of $\Delta\tau$, the probe beam will encounter the THz peak at the second antenna (Ant. 2) only after the delay line has scanned an extra time interval equal to $\Delta\tau$, anticipating the arrival of the THz pulse arrival at the chip. However, contrary to the first case, after crossing Ant. 2, the probe beam will move towards Ant. 1 which has already started resonating, thus further interacting with its THz near-field, yet during a delayed evolution of the THz oscillations. Similar considerations can be made for all the subsequent antennas. Supplementary Fig. 11 shows the temporal order of the emergence of the THz

transients corresponding to each antenna as experienced by the probe beam. The black curve represents the resultant phase modulation transient imparted to the probe beam due to the linear superposition of all antenna contributions. We point out that the final waveform exhibits an oscillating behavior with each local maximum corresponding to the arrival time of the probe beam at each antenna. Indeed, the case represented in this figure corresponds to the device explored in the main manuscript and it has to be ascribed to the fact that the antenna resonance is detuned from the phase-matching frequency of the array.

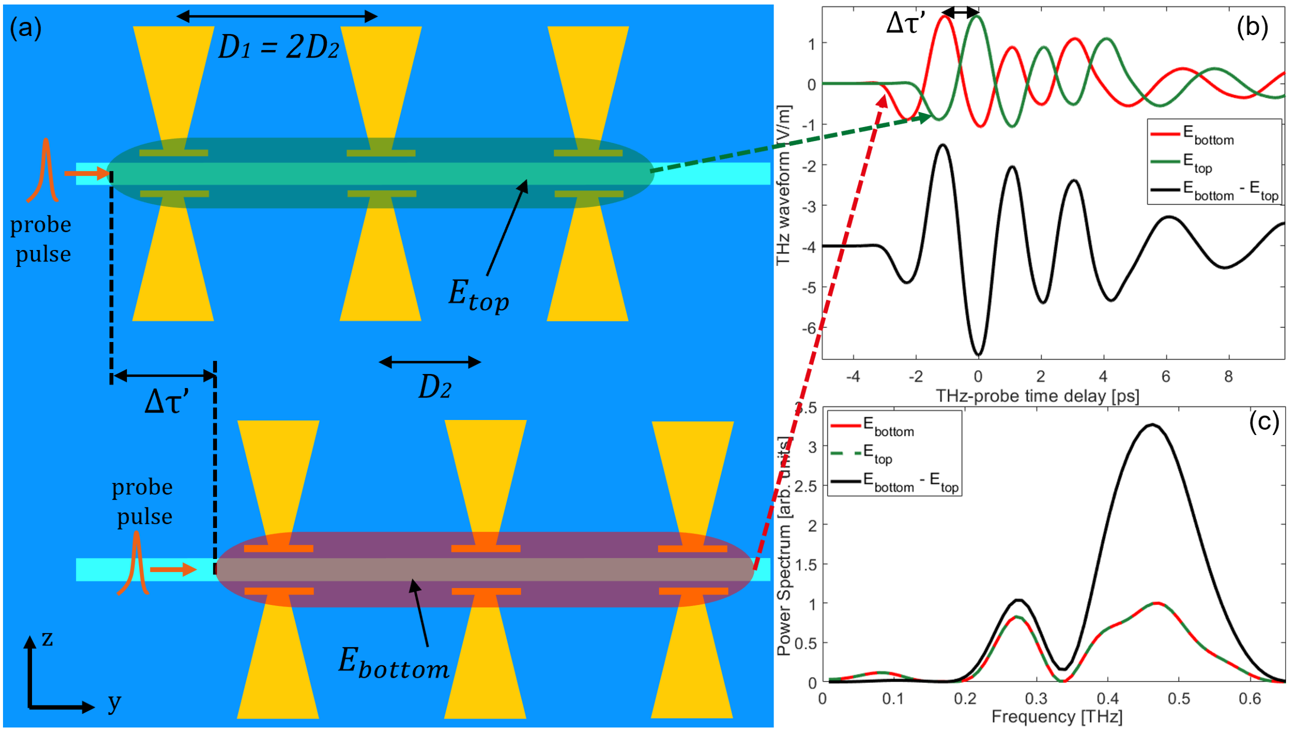

Supplementary Fig. 12: Time diagram of the THz-induced intensity modulation of a probe beam imparted by the interference of two displaced arrays of antennas. (a) Sketch depicting two arrays of antennas, each running along one arm of a MZI device (not shown in the figure). Both top (red halo) and bottom (green halo) arrays generate the same THz-induced phase modulation transient (as that in Supplementary Fig. 11) that modulates each corresponding sub-probe. The different color of the halo enveloping each array indicates that the THz near-field established across the corresponding arm only differs from a phase factor. (b) The longitudinal displacement between the two arrays leads to a mutual temporal delay $\Delta\tau' = \Delta\tau/2$ between the arrival times of the two sub-probe and in turn of the corresponding time-dependent phase modulation (red and green solid lines for the top and bottom array, respectively). This leads to a specific interference of the two sub-probes, which intensifies and converts the total phase modulation into the amplitude modulation of the output probe beam (black solid line). The latter is vertically shifted for clarity only. (c) Fourier Transforms of the curves in (b). The spectrum of the interference transient reveals a significant enhancement of the component at the phase-matching frequency of the array (i.e., $1/\Delta\tau$, black solid line) compared to the case of a single array (red and green lines). Curves are normalized to the maximum of the interference spectrum (black solid line).

Consequently, any time the probe beam is temporally aligned to the THz peak of a specific antenna, the remaining oscillations of the THz transients in neighboring antennas do not significantly contribute to its phase modulation. This fact allows us to precisely associate each waveform peak to the spatial position of a specific antenna along the array, which is the key feature enabling the beam profiling capability provided by our device, as described in the main text. The time diagram used to explain the order of interaction between the probe beam and each antenna within an array can be used to interpret the formation of the total phase modulation transient acquired by the probe beam when the two arms of the MZI interfere, as depicted in Supplementary Fig. 12. In particular, panel (a) shows the case of a device where the array on the bottom arm is displaced in the forward direction by a temporal delay equal to $\Delta\tau'$ compared to that on the top arm. By considering the entire array as a single entity that gives rise to an overall phase modulation transient as that seen in Supplementary Fig. 11b, we can draw the plot shown in Supplementary Fig.

12b. Due to the array displacement being equal to half of that between consecutive antennas (i.e., $\Delta\tau' = \Delta\tau/2$), each sub-probe in the interferometer will experience a phase modulation of mutual opposite polarity. As derived in Eq. 7, the superposition of the electric field of the two sub-probes at the output of the interferometer results in the difference between the two individual phases. Therefore, the final phase modulation transient imparted to the recombined probe beam is the difference between the single array, which corresponds to a positive adding up of the two contributions, due to the inherent opposite polarity. It is worth noticing that the interference between the two arms preserves the time position of each peak in the final waveform, and thus, in turn, the bijective mapping with the spatial location of each single antenna along both arrays. Finally, Supplementary Fig. 12c shows the power spectra associated with the single array and double displaced cases. We note that the interference between the two arms enhances the component at the phase-matching frequency, which is the dominant oscillation cycle in the corresponding waveform. This further filtering action due to the waveforms interference improves the visibility of each waveform peak, and in turn the beam profiling capability.

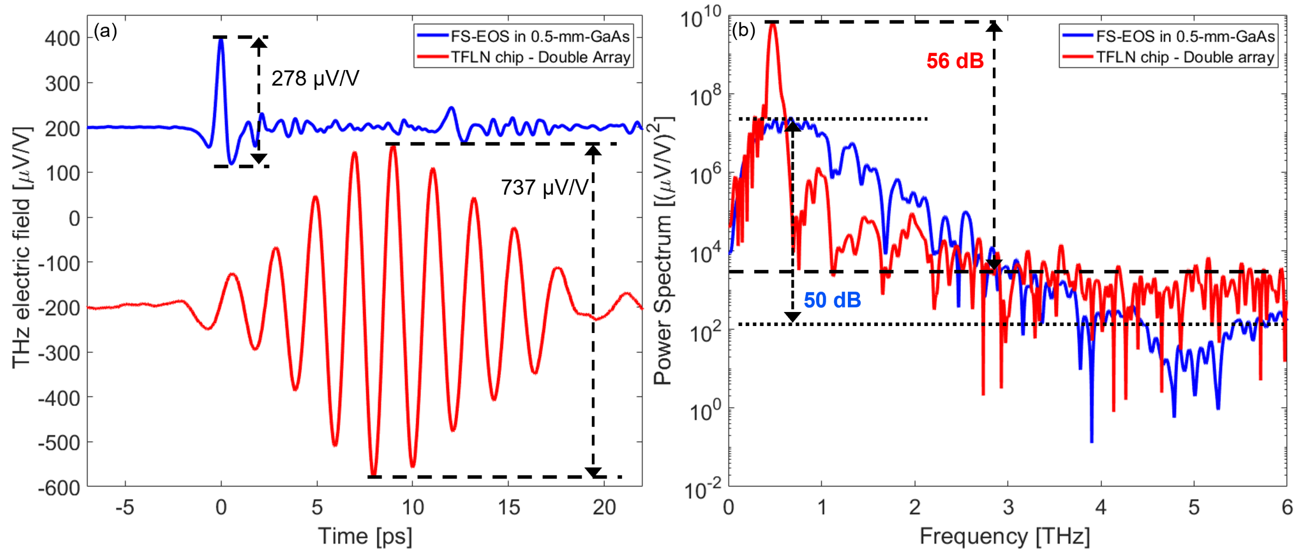

Supplementary Fig. 13: The time and spectral responses of FS-EOS and TFLN chips to a broadband THz test pulse. Comparison between THz detection carried out via conventional FS-EOS in a 0.5-mm-GaAs crystal and the TFLN chip, both probed at a 1550 nm wavelength, in terms of time traces (a) and corresponding spectra (b). FS-EOS provides relatively broadband spectral response and corresponding shorter temporal transients, owing to a larger collinear phase-matching bandwidth, which depends on the crystal properties at THz and optical frequencies. The TFLN chip provides very high sensitivity in a relatively narrow linewidth centered at the built-in phase-matching frequency of the antenna array. Albeit the single-ended nature of the optical detection and the spatially-limited THz-optical pulse interaction for the TFLN chip case, the associated spectral dynamic range is larger than that of the FS-EOS case.

6 Supplementary Note 6: Comparison between Free-Space Electro-Optic Sampling technique and the electro-optic detection in TFLN chips

In Supplementary Fig. S9, we provide a direct comparison, in terms of both time-domain waveforms (a) and corresponding spectra (b), between THz wave detection carried out via conventional free-space electro-optic sampling (FE-EOS) in a 0.5-mm-GaAs crystal and our TFLN chip featuring a double array of 9 antenna each. In both cases, the probe beam has a nominal wavelength of 1550 nm and it travels through the same optical fiber, thus ensuring that the probe pulse duration is quite similar in both schemes. The probe power reaching the photodetector is 0.5 mW on each channel of the balanced photodetector in the case of FS-EOS, whereas it accounts for 0.2 mW in the single channel of the same photodetector utilized to acquire the optical probe coupled out from the TFLN chip. The input THz electric field strength is fixed at 58 V cm^{-1} and 4.5 V cm^{-1} for the case of FS-EOS and TFLN chip,

respectively. As we can see from Supplementary Fig. S9, the quasi single-cycle THz waveform recorded via FS-EOS lasts slightly less than 2 picoseconds with a modulation peak-to-peak modulation approaching 300 μV/V. This corresponds to a power spectrum that extends beyond 4 THz (Supplementary Fig. S9b), where the noise floor lies around 50 dB below the spectrum peak. In the case of the TFLN chip, the subsequent probing of the antenna array gives rise to a recorded waveform exhibiting a multicycle shape. As mentioned in the main text, the number of periods featuring the entire waveform is equal to the number of antenna pairs (i.e., 9 in this case), while the period length is equal to the inverse of the phase-matching frequency underlying the antenna array. A modulation peak-to-peak value exceeding 700 μV/V is observed. In the frequency domain, the spectrum associated to the TFLN chip clearly reveals the strong effect of the selection rule implied by the quasi-phase-matching mechanism operated by the double array. We observe a strong, relatively narrow peak at around 480 GHz. Away from this phase matching frequency, signals are suppressed by more than 40 dB (e.g. around 730 GHz). 100 GHz away from this phase matching frequency, signals are suppressed by more than 20 dB. Spectral peaks at higher frequencies could be ascribed to either higher order modes induced in the resonating antennas or imperfect balancing of the two arms of the interferometer. It is worth noticing that the noise floor for the TFLN chip case starts at frequencies above 2 THz with a level around 10 dB higher than that of the FS-EOS case. Yet, the spectral dynamic range accounts for 56 dB, slightly higher than the free-space approach despite the lower THz strength utilized as an input. This means that in the TFLN chip case, the high field enhancement within the 3.3-μm-large and 60-μm-long gap greatly compensate for the naturally ultra-short THz-probe spatio-temporal overlap affecting a cross-propagation geometry. Moreover, it should be noted that while the FS-EOS is inherently a differential technique, which benefits for a further common noise rejection mechanism, detection based on the TFLN chip is currently operated according to a single-ended configuration. This is the main reason behind the higher noise floor observed in this case. Noteworthy, we envision the possibility to further engineer the output waveguide of our devices, such as through the adoption of an X-coupler configuration, potentially enabling for the implementation of an integrated differential scheme, similar to that underlying free-space electro-optic sampling.

7 Supplementary Note 7: Dependence of the frequency response of the device on the angle of incidence of the input THz beam

In order to predict the spectral behavior of the device under illumination of a THz beam incident at an arbitrary angle, we have developed an extensive analytical model, which we provide in the following text. For the sake of simplicity, we will start by distinguishing between two main configurations, i.e., the horizontally and vertically tilted THz beams, and finally we will generalize to the case of an arbitrary angle of incidence. Likewise, each antenna element is initially treated as an isotropic radiator (i.e., equally emitting along every direction in space), whereas its angular-dependent radiation pattern will be used to particularize the results obtained for the isotropic array.

Horizontally tilted THz beam illumination.

Figure 14a represents the case of a horizontally tilted THz beam illumination. In this case, the THz beam forms an angle θ with respect to the vector normal to the device, and it is inclined in such a way as to induce a delayed illumination of the two arms of the interferometer, as indicated in the two-dimensional sketch of Fig. 14b. In this configuration, the first important aspect to take into account is the polarization of the incoming THz beam. Under orthogonal incidence, the detection efficiency is maximized when the THz polarization is aligned with the antenna orientation (i.e., along the z-axis). However, under a tilted angle of incidence, the THz beam can be

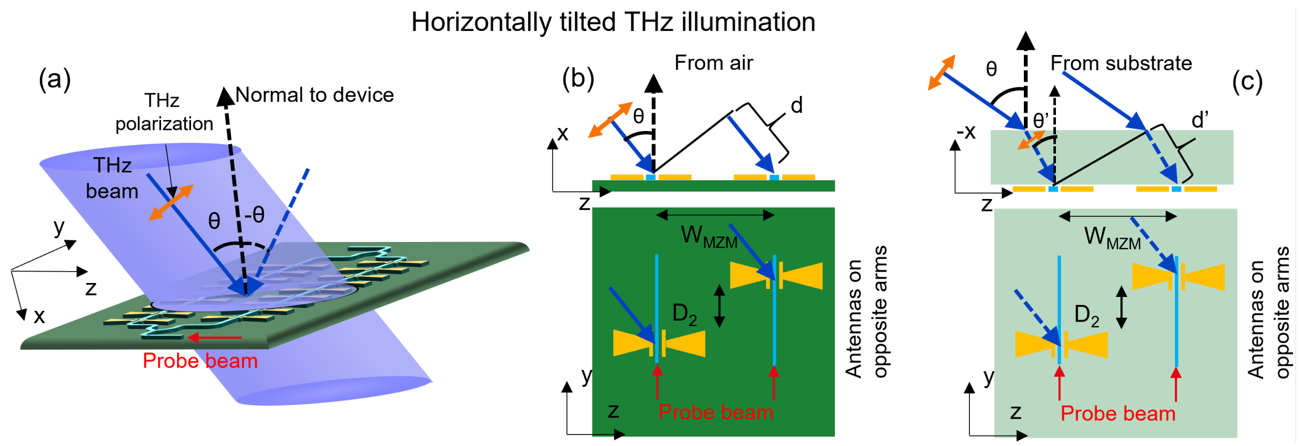

Supplementary Fig. 14: TFLN device under illumination of an horizontally tilted THz beam. **a** Perspective view of the THz beam forming an angle with the vector normal to the device that leads to an nonuniform illumination of the two arms of the interferometer. **b** Top view of the configuration in (a) for the case of the THz beam impinging from (b) the air and (c) the silicon substrate.

decomposed into two polarization components: one still parallel to the antenna direction (z-axis) and another along the longitudinal direction (x-axis). The latter component barely couples to the antenna and can be neglected, thus effectively contributing to the whole THz loss of the device. As shown in the manuscript, in the case of an orthogonally incident THz beam, the spacing between antennas on opposite arms (D2 = D1/2) is chosen in order for the probe beam to sense the THz near field at opposite polarities of the THz cycle. Here, the inclination of the THz beam alters such a built-in timing (indicated as Δt_2 in the main manuscript), due to the extra time delay δt taken by the THz beam to travel the distance d and reach the second arm (see Fig. 14(b)). This extra time delay can be calculated using the following formula:

$$\delta t_{HT} = \frac{n_{THz}^{air} W_{MZM} \sin\theta}{c} \tag{20}$$

where the subscript HT stands for "horizontal tilt", n_{THz}^{air} is the THz refractive index of air and c is the speed of light in vacuum. The reason why we explicitly keep $n_{THz}^{air} = 1$ in Eq. 20 will be clear afterwards, when we will consider the illumination from the substrate side, and n_{THz}^{air} will be replaced by n_{THz}^{Si}. Now we can correct the expression of Δt_2 as:

$$\Delta t_2'(\theta) = \Delta t_2 \pm |\delta t_{HT}| = \frac{n_g D_1}{2c} \pm \left| \frac{n_{THz}^{air} W_{MZM} \sin\theta}{c} \right|. \tag{21}$$

The plus/minus sign in the Eq. 21 depends on the reciprocal orientation between the THz beam inclination and propagation direction of the probe beam. In the case depicted in Fig. 14a, if we assume that the probe beam is injected from the lower side of the interferometer, we can take the minus sign in Eq. 21. Consequently, the angle θ spans the range $[-90°, 90°]$ in such a way that positive values correspond to a reduced $\Delta t_2'(\theta)$, whereas negative values increases the value of $\Delta t_2'(\theta)$. By plugging Eq. 21 into Eq. 1 of the main manuscript, we can then write the dependence of the frequency response of the entire device on the impinging angle θ:

$$\frac{\Delta I(f)}{I_{out}^{Q} E_{ant}(f)} \propto -2i \frac{\sin(\pi f N_{ant} \Delta t_1)}{\sin(\pi f \Delta t_1)} e^{i\pi f[\Delta t_1 (N_{ant}-1) + \Delta t_2'(\theta)]} \sin(\pi f \Delta t_2'(\theta)) \tag{22}$$

Finally, taking the modulus of Eq. 22 and using Eq. 21, we achieve:

$$|T_{MZI}(f,\theta)| = 2 \frac{\sin(\pi f N_{ant} \Delta t_1)}{\sin(\pi f \Delta t_1)} \sin\left(\pi f \left[\frac{n_g D_1}{2c} - \frac{n_{THz}^{air} W_{MZM} \sin\theta}{c}\right]\right) \tag{23}$$

A quick inspection of Eq. 23 reveals that this type of THz beam inclination does not influence the array periodicity, but rather it affects the interference between the two arrays of antennas on opposite arms. This effect is shown in Fig. 15a and b, where the device response as expressed in Eq. 23 is plotted for different angles of incidence. We note

that for the case of positive angles (a), the original phase-matching frequency (i.e., 487 GHz) does not exhibit any shift, but the corresponding sensitivity greatly changes. Moreover, the frequency response at even harmonics becomes non-null and even dominant in some cases, in stark contrast with the orthogonal case. The case of negative angles (b) appears somewhat more complex. The overall trend is similar to that of the positive counterpart, yet the peak frequency slightly shifts with respect to the original phase-matching frequency. Such a shift is even more pronounced at the higher harmonics. However, a more peculiar effect occurs when the angle approaches a critical value:

$$\theta_{cr} = -\arcsin\left(\frac{n_g D_1}{2 n_{THz}^{air} W_{MZM}}\right) = -27.39^\circ \tag{24}$$

Under this angle of incidence, the overall delay between two arms become synchronous with the time taken by the probe beam to travel between two antennas on the same array, that is:

$$\Delta t_2'(\theta_{cr}) = \frac{n_g D_1}{2c} - \frac{n_{THz}^{air} W_{MZM}}{c}\sin(\theta_{cr}) = \frac{n_g D_1}{2c} + \frac{n_{THz}^{air} W_{MZM}}{c}\frac{n_g D_1}{2 n_{THz}^{air} W_{MZM}} = \frac{n_g D_1}{c} = \Delta t_1. \tag{25}$$

Therefore, at the critical angle, the rate at which the two arms interfere ($= 1/\Delta t_2'$) equals the phase-matching frequency ($= 1/\Delta t_1$) of the array. As such, the two resonances couple and give rise to a seemingly unbounded spectral response observed in Fig. 15b (green curve). This can be easily predicted by using the results of Eq. 25 into Eq. 22, and obtaining:

$$|T_{MZI}(f, \theta_{cr})| = 2\frac{\sin(\pi f N_{ant}\Delta t_1)}{\sin(\pi f \Delta t_1)}\sin\left(\pi f \Delta t_2'(\theta_{cr})\right) = 2\frac{\sin(\pi f N_{ant}\Delta t_1)}{\sin(\pi f \Delta t_1)}\sin\left(\pi f \Delta t_1\right) = 2\sin(\pi f N_{ant}\Delta t_1) \tag{26}$$

Equation 26 indicates that the double array suddenly becomes greatly sensitive to a countably infinite set of frequency components, as shown in Fig. 15b. Interestingly, we note that for a positive incidence angle equating in modulus θ_{cr}, the mutual delay between the two arrays becomes:

$$\Delta t_2'(-\theta_{cr}) = \frac{n_g D}{2c} - \frac{n_{THz}^{air} W_{MZM}}{c}\sin(-\theta_{cr}) = 0. \tag{27}$$

leading to:

$$|T_{MZI}(f, -\theta_{cr})| = 2\frac{\sin(\pi f N_{ant}\Delta t_1)}{\sin(\pi f \Delta t_1)}\sin\left(\pi f \Delta t_2'(-\theta_{cr})\right) = 0. \tag{28}$$

This means that the device exhibits an identically null amplitude response for a positive angle equal to θ_{cr}. This is because the two probe beams undergo the same phase modulation on each arm, thus simply adding up upon recombination at the output of the interferometer. In this condition, the device operates as a purely phase modulator and THz detection and cannot be carried out with a simple photodetector.

It should be noted that the complete spectral behavior of the entire device involves convolution with the frequency response of a single antenna. Therefore, we calculated the spectrum of the retrieved THz spectrum at the end of the interferometer and generated the plots in Fig.15c and d for the negative and positive angles, respectively. We note that some of the features observed for the spectral response of the double array (e.g., decrease of the amplitude at the built-in f_{PM} component and appearance of smaller peaks for negative angles) are indeed transferred to the output THz spectrum, yet with a small impact on the overall spectral response. As a detailed summary, Fig. 15e displays how the peak frequency of the spectra in plots (c) and (d) moves away from the nominal $f_{PM} = 487$ GHz as a function of the titling angle. While the frequency deviation is rather negligible for positive angles, the trend shows an interval of angles where the antenna resonance ($f_{ant} = 360 GHz$) takes over, due to the coupling effect occurring closely around the critical angles. Finally, we note that the device remains tuned at the original f_{PM} within an interval of angles as wide as $\Delta\theta_{FWHM} = 20^\circ$ (full-width-half-maximum) (see Fig. 15e).

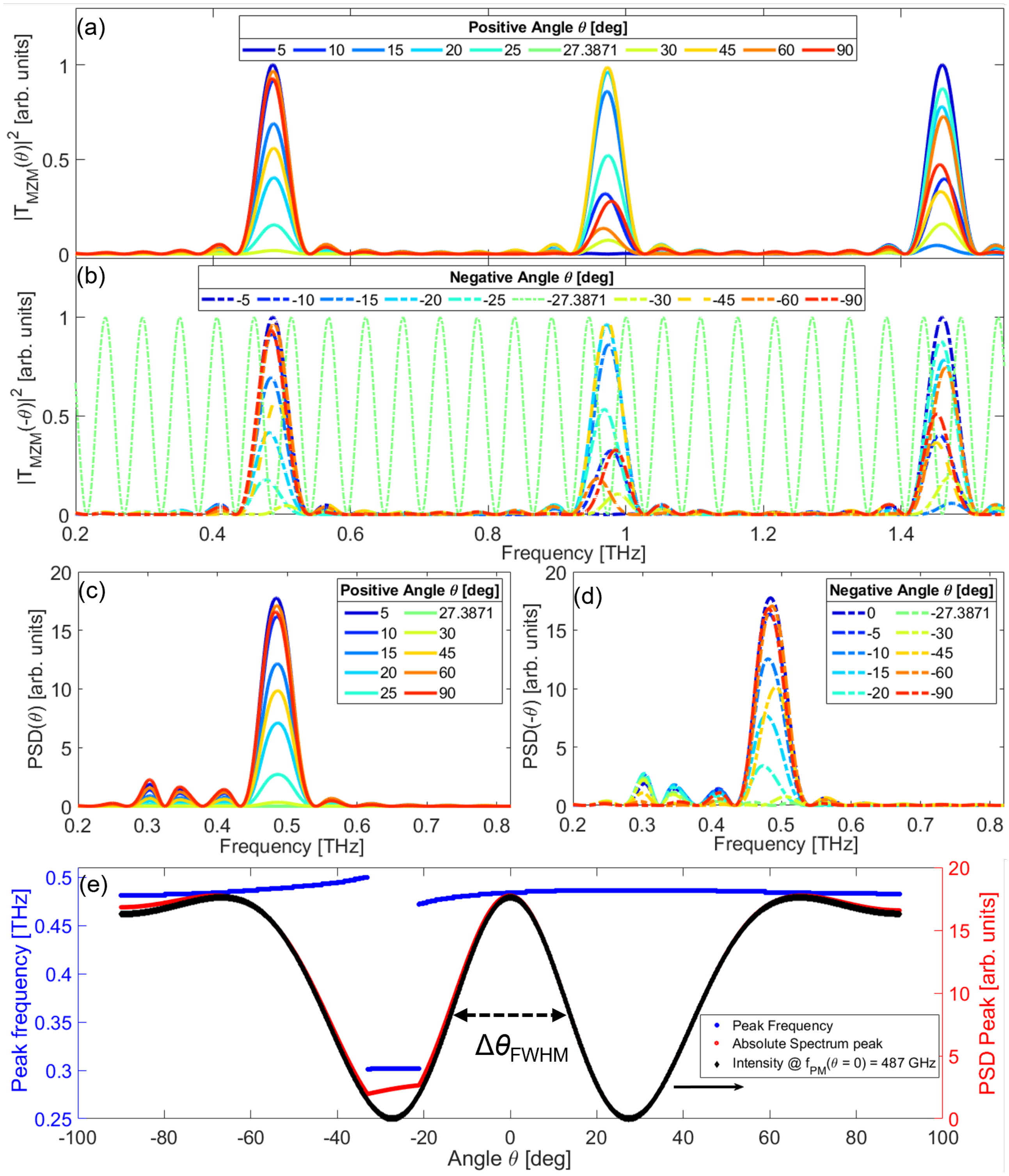

Supplementary Fig. 15: MZI response as a function of the angle of incidence for an horizontally tilted THz beam. Transfer function of the MZI device for the case of **(a)** positive and **(b)** negative values of the angle θ. Curves are normalized to their own maxima. Calculated THz spectra retrieved at the output of the MZI for the case of **(c)** positive and **(d)** negative angles θ, corresponding to the case in **(a)** and **(b)**, respectively, convoluted with the spectral response of the single antenna. **(e)** Peak frequency of the reconstructed THz spectra (blue line) and corresponding peak amplitude (red line) as a function of the angle of incidence θ. The amplitude intensity of the component at the built-in $f_{PM}(\theta = 0) = 487$ GHz (black line) overlaid to the red curve points out the robustness of the device response against the THz beam tilt.

Figure 14c shows the case of THz beam illumination coming from the silicon substrate side. In this case, the THz

beam first undergoes refraction at the air/silicon interface according to Snell's law:

$$n_{THz}^{air}\sin\theta = n_{THz}^{Si}\sin\theta' \tag{29}$$

Inside the silicon substrate, the refracted THz ray bends towards the vector normal to the surface, thus the geometrical path difference between the two arms of the interferometer is:

$$d' = W_{MZM}\sin\theta' = W_{MZM}\frac{n_{THz}^{air}\sin\theta}{n_{THz}^{Si}} = \frac{d}{n_{THz}^{Si}}. \tag{30}$$

However, since the THz path within the silicon substrate is still $n_{THz}^{Si}d' = d$, the phase retardation induced in the two arms follows the exact same equations deduced for the case of illumination from the air side. Therefore, we conclude that the overall spectral behavior of the device is irrespective of the illumination side.

Vertically tilted THz beam illumination.

Figure 16a shows a perspective view of the case of a vertically tilted THz beam illumination. In this case, the THz beam forms an angle α with the vector normal to the device and it is inclined in such a way that, using the propagation direction of the probe beam as a reference, the THz beam encounters the lower antennas at earlier times than the upper ones on both arms (Fig. 16b). It should be noted that the delay between the two arms, i.e., Δt_2, is accordingly modified since the relation $\Delta t_2 = \Delta t_1/2$ still holds. Under this type of THz beam inclination, the antennas located on the same arm do not resonate synchronously. This has to be ascribed to the fact that the THz beam has to propagate a path difference between two consecutive antennas equal to $b = D_1\sin\alpha$, as indicated in Fig. 16b. The extra time delay between contiguous antennas can be calculated as:

$$\delta t_{VT} = \frac{n_{THz}^{air}D_1\sin\alpha}{c} \tag{31}$$

where the subscript VT stands for "vertical tilt". Therefore, the time interval taken by the probe beam to move between two antennas excited by the THz beam is now corrected as:

$$\Delta t_1'(\alpha) = \Delta t_1 \pm |\delta t_{VT}| = \frac{D_1 n_g}{c} \pm \left|\frac{n_{THz}^{air}D_1\sin\alpha}{c}\right| = \frac{D_1}{c}(n_g \pm |n_{THz}^{air}\sin\alpha|) \tag{32}$$

Similarly to the previous case, the plus/minus sign depends on the reciprocal orientation of the probe and THz beam prorogation direction. For the case depicted in Fig. 16, we take the minus sign and consider "positive" the values of α that effectively decreases the time interval $\Delta t_1'(\alpha)$. By plugging Eq. 32 into Eq. 1 of the main manuscript and taking the modulus, we obtain:

$$|T_{MZI}(f,\alpha)| = 2\frac{\sin\left(\pi f N_{ant}\frac{D_1}{c}(n_g - n_{THz}^{air}\sin\alpha)\right)}{\sin\left(\pi f\frac{D_1}{c}(n_g - n_{THz}^{air}\sin\alpha)\right)}\cdot\sin\left(\pi f\frac{D_1}{2c}(n_g - n_{THz}^{air}\sin\alpha)\right) \tag{33}$$

From Eq. 33 we can infer that this type of THz beam inclination affects the built-in phase accumulation induced into the probe beam, by altering the mutual arrival of the probe and THz beam to each antenna. At first glance, we could expect that the phase-matching frequency $f_{PM}'(\alpha)$ for the single array is now a function of the angle α according to:

$$f_{PM}'(\alpha) = \frac{1}{\Delta t_1'(\alpha)} = \frac{c}{D_1(n_g - n_{THz}^{air}\sin\alpha)} \tag{34}$$

However, Fig. 17 showcases how the trends described by Eq. 33 for positive (panel (a)) and negative (panel (b)) angles α differ quite significantly. More in detail, the spectrum shifts towards higher frequencies as the angle spans larger positive values (Fig. 17a). However, the spectral sensitivity decreases dramatically with the increasing angle, even though the linewidth around the peak slightly stretches, indicating a somewhat broader response compared to

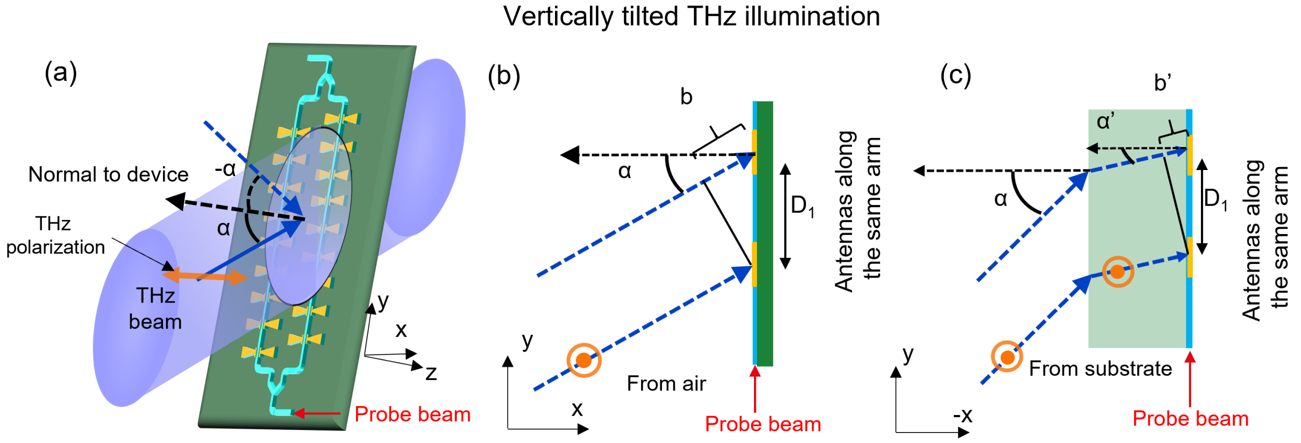

Supplementary Fig. 16: TFLN device under illumination of a vertically tilted THz beam.(a) Perspective view of the THz beam forming an angle α with the normal to the device that leads to the delayed illumination of antennas located on the same arm of the interferometer. Lateral view when the THz beam impinges from **(b)** the air and **(c)** the silicon substrate.

the orthogonal case. For negative values of α (Fig. 17b), the spectral response exhibits a red shift with slower pacing compared to the blue shift in (a), along with a corresponding small amplitude change. In addition, the second-harmonic component emerges from the background reaching amplitude values similar to the first harmonic. The actual THz electric field spectra simulated at the end of the interferometer depict how the angle α profoundly changes the overall behavior of the device. As shown in Fig. 17c, the spectral response of the single antenna superimposed to the curve of Fig. 17a heavily damps THz detection for positive values, since $f'_{PM}(\alpha)$ deviates ever so much from the eigen resonance of the antenna. On the other hand, the effect is reversed for the case of negative values Fig. 17d, since the reconstructed THz spectra show an ever-increasing spectral peak as the incidence approaches the grazing condition (i.e., 90°). This is because $f'_{PM}(\alpha)$ approaches the resonance of the single antenna, further boosting the efficiency of the THz-induced phase modulation of the probe beam. Finally, Fig. 17e summaries the results of the upper panels, showing how the THz spectral intensity at the built-in phase-matching frequency ($f'_{PM}(\alpha = 0) = f_{PM}$ $= 487$ GHz) quickly decreases to a nearly zero value for angle deviation larger than $\Delta\alpha_{FWHM} = \pm 10°$. We also note that the peak frequency of the reconstructed THz spectrum matches the values predicted by the formula in Eq. 34 only for negative angles α. For positive values, the trend largely diverges from that predicted by this simple model and even exhibits two strong discontinuities. The first deviation occurs when the following condition is satisfied:

$$\begin{aligned}\Delta t'_1(\alpha_{cr1}) &= \frac{D(n_g - n^{air}_{THz}\sin\alpha_{cr1})}{c} = \frac{3}{4}\Delta t'_1(\alpha = 0)\\ n_g - n^{air}_{THz}\sin\alpha_{cr1} &= \frac{3n_g}{4} \Rightarrow \sin\alpha_{cr} = \frac{n_g}{4} \Rightarrow \alpha_{cr1} = \arcsin\frac{n_g}{4} = 35.1°.\end{aligned} \tag{35}$$

This condition is associated to a relatively abrupt drop of the amplitude. The second discontinuity happens when:

$$n_g - n^{air}_{THz}\sin\alpha_{cr2} = \frac{\pi}{2} \Rightarrow \alpha_{cr2} = \arcsin\left(n_g - \frac{\pi}{2}\right) = 46.8°. \tag{36}$$

In this second case, the amplitude becomes identically equal to zero once again. However, the impact of this effect is quite insignificant since the response is already very weak around the second critical angle.

Finally, Fig. 16c shows the case of a THz beam incoming from the silicon substrate. This situation is the analogue of what has been seen in Fig. 14c. Because of refraction, Snell's law allows to write:

$$n^{air}_{THz}\sin\alpha = n^{Si}_{THz}\sin\alpha' \tag{37}$$

Then, the extra THz path delay accumulated upon reaching the array is:

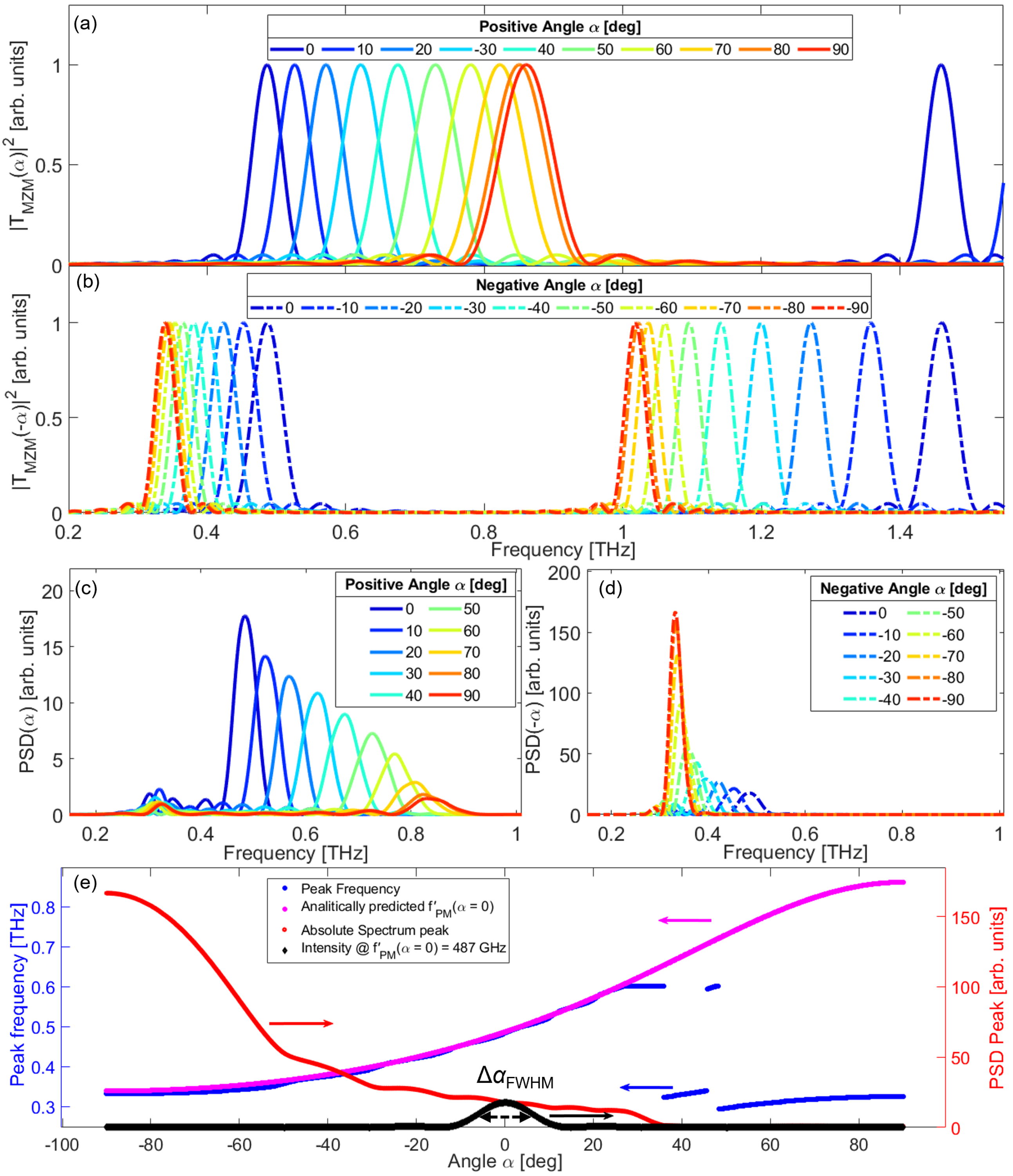

Supplementary Fig. 17: MZI response as a function of the angle of incidence for a vertically tilted THz beam. Transfer function of the MZI device for the case of **(a)** positive and **(b)** negative values of the angle α. Curves are normalized to their own maxima. Calculated THz spectra retrieved at the output of the MZI for the case of **(c)** positive and **(d)** negative angles α, corresponding to the case in **(a)** and **(b)**, respectively, convoluted with the spectral response of the single antenna. **(e)** Peak frequency of the reconstructed THz spectra (blue line) and corresponding peak amplitude (red line) as a function of the angle of incidence θ. The theoretical trend of the angle-dependent phase-matching frequency is drawn with a purple line. The amplitude intensity of the component at the built-in $f'_{PM}(\alpha = 0) = f_{PM} = 487$ GHz (black line) overlaid to the red curve points out the robustness of the device response against the THz beam tilt.

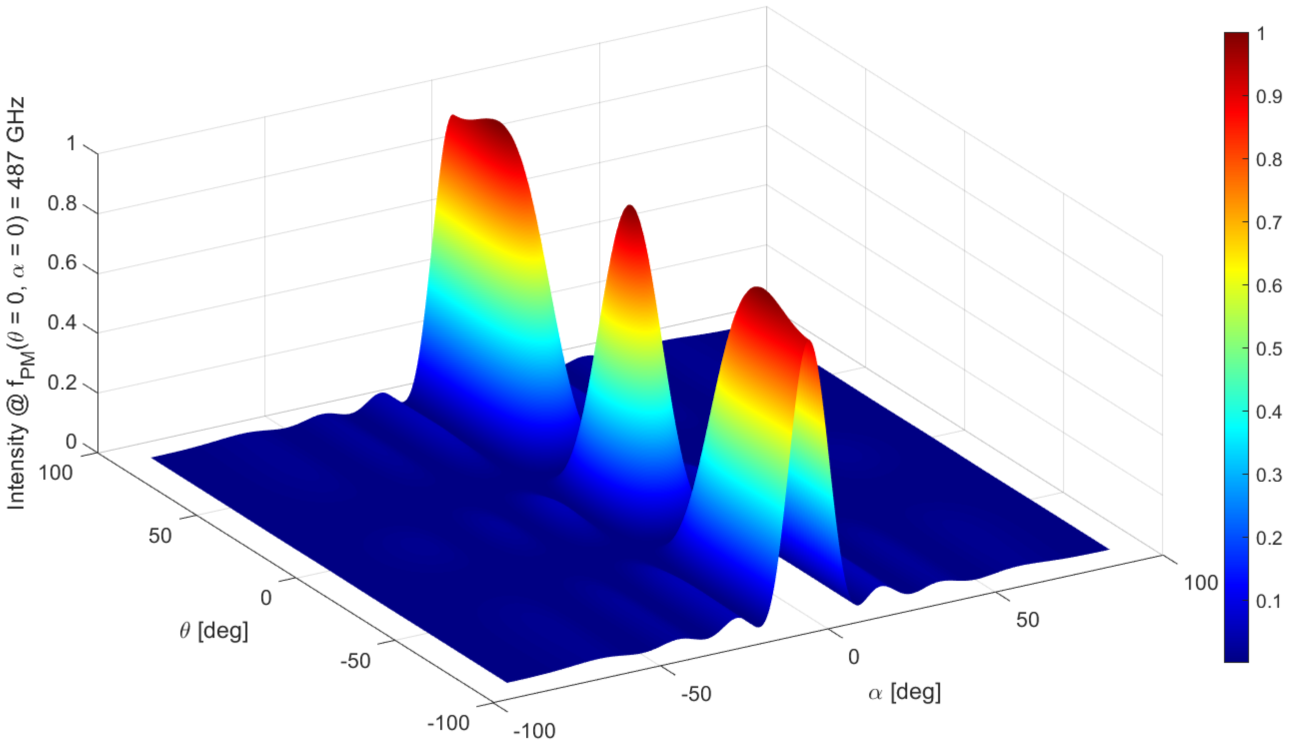

Supplementary Fig. 18: Intensity of the built-in (nominal) phase-matching frequency $f'_{PM}(\theta = 0, \alpha = 0) = f_{PM} =$ 487 GHz as a function of an arbitrary tilted THz beam, impinging from the air side onto an array of isotropic antenna elements. The 3D plot reports dependence of the original phase-matching frequency on all combinations of angles θ and α.

$$\delta t'_{VT} = \frac{n_{THz}^{Si} b'}{c} = \frac{n_{THz}^{Si} D_1 \sin\alpha'}{c} = \frac{D_1 n_{THz}^{air} \sin\alpha}{c} = \delta t_{VT} \tag{38}$$

Therefore, we conclude that the overall trend is irrespective of the illumination side and the same equations applies to this case. In general, we note that an arbitrary oblique incidence can be decomposed in the two cases examined above, by projecting the THz beam into a horizontally and vertically tilted beam components. To this end, we report a 3D plot of the device response at the built-in $f'_{PM}(\alpha = 0, \theta = 0) = f_{PM}$ in Fig. 18, for all combinations of angles θ and α.

Radiation pattern of the single antenna.

As mentioned previously, the results of the model presented so far are valid only for the simple case of an antenna array made of isotropic elements. However, this is not usually the case. Therefore, in order to account for the specific radiation pattern of each single antenna, we simulated its angular dependence through CST simulations. Figure 19 shows the projections of radiation pattern of the antenna on the planes xz (Fig. 19a) and yx (Fig. 19c), where the angles θ and α span the $[-90°, 90°]$ range, respectively. Both plots are evaluated at the nominal phase-matching frequency ($f_{PM} = 487$ GHz) of the array. In the first instance, we consider the THz beam impinging from the air side of the chip. This corresponds to the area highlighted in red and blue in panels (a) and (c), respectively. Figures 19b and d depict the dependence of the normalized modulus of the THz electric field collected by the antenna as a function of the angles θ and α, respectively. By combining these results with the isotropic array case, we can modify the 3D plot presented in Fig. 18, which now becomes as shown in Fig. 20a. Therefore, the main effect of the specific radiation pattern featuring the single antenna is the attenuation of the spectral response of the device beyond the ranges of values $\Delta\theta_{FWHM}$ and $\Delta\alpha_{FWHM}$, whereas the latter two angular detuning remain essentially unaffected.

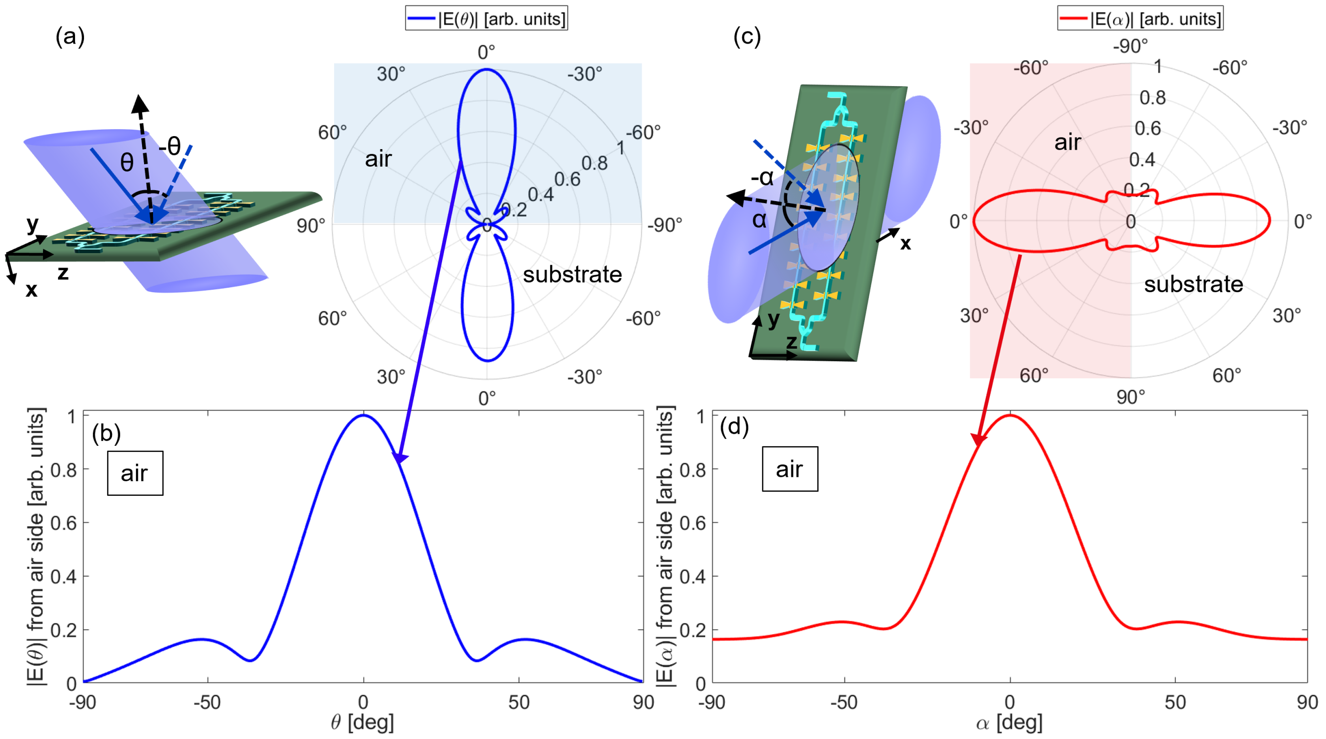

Supplementary Fig. 19: Radiation pattern of a single bow-tie antenna at f_{PM} = 487 GHz. Polar plots showing the acceptance angle of the antenna in the (a) xz plane corresponding to variation of the angle θ and (c) xy plane corresponding to changes of the angle α. The area highlighted in blue and red in both polar plots indicate the air side. Sketches of devices at the corresponding cut plane are shown to identify the direction of the incoming THz beam. Normalized modulus of the THz electric field as a function of the angles (b) θ and (d) α, corresponding to the air side of the polar plots in (a) and (c), respectively.

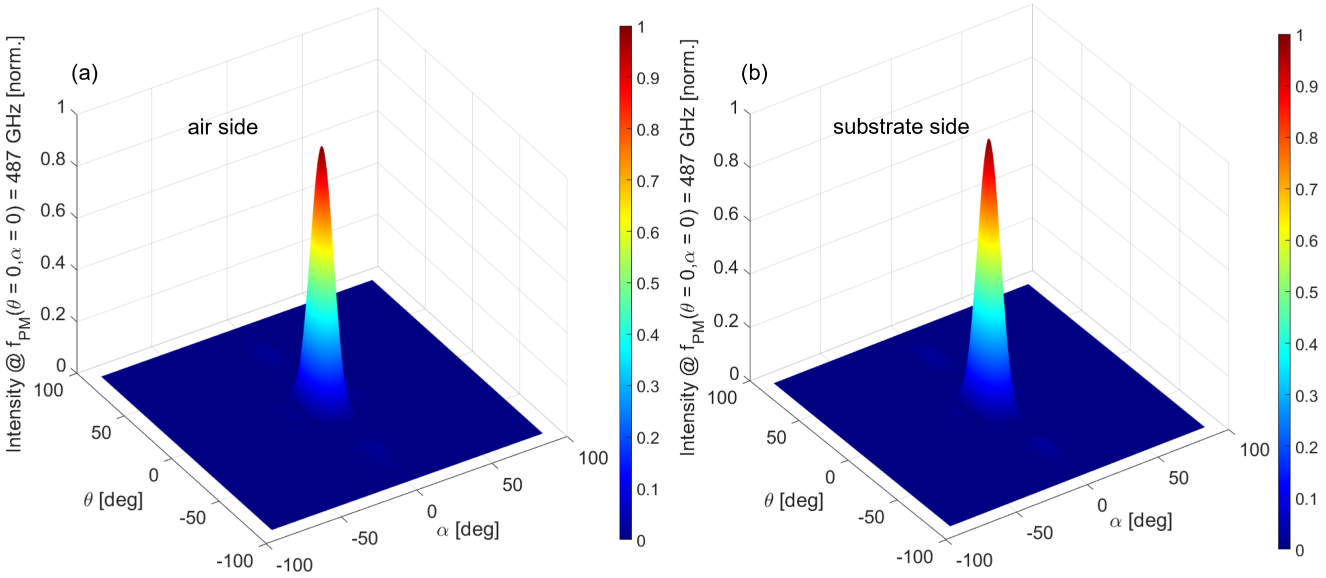

Supplementary Fig. 20: Intensity of the built-in phase-matching frequency component $f'_{PM}(\theta = 0, \alpha = 0) = f_{PM}$ = 487 GHz as a function of an arbitrary tilted THz beam. The 3D plot is the result of the isotropic response of the array combined with the radiation pattern of the antenna towards the (a) air side and (b) silicon substrate side. In both panels, the curve is normalized with respect to its own maximum.

This makes the entire device much less sensitive to disturbs owing to stray radiation incoming at random angles, also mitigating vulnerability to jamming attacks. The same procedure can be followed to determine the complete

spectral response for the case of a THz beam impinging from the side of the substrate. This is shown in Fig. 20b, which reveals a regime of operation very similar to the air case.

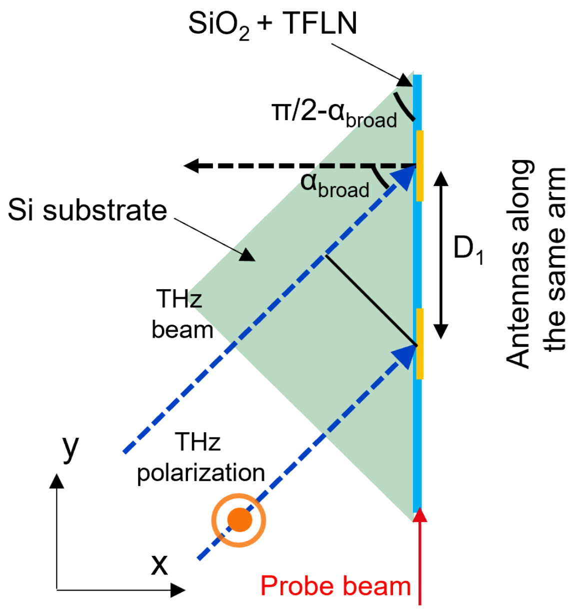

Supplementary Fig. 21: Sketch of the chip geometry for broadband THz detection with antenna array. By etching the rear facet of the silicon substrate at the specific angle $\pi/2 - \alpha_{broad}$, a THz beam hitting the device at the complementary angle of incidence (α_{broad}, measured at the plane of the antenna array), would orthogonally penetrate the substrate, avoiding angular refraction and thus undergoing the exact amount of phase retardation that leads to broadband detection.

Broadband THz detection using antenna arrays.

Finally, we would like to mention that a way to truly achieve broadband THz detection with an antenna array (or double, as in our case) is to impose that the transmission of the MZI is identically equal to 1 at any THz frequency in Eq. 33. In other words, the time delay $\Delta t_1'(\alpha)$ must be set to zero. Therefore, we find out that:

$$|T_{MZI}(f,\alpha)| = 1 \ \forall f \ \Rightarrow n_g - n_{THz}^{medium} \sin\alpha = 0 \Rightarrow \alpha_{broad} = \arcsin\left(\frac{n_g}{n_{THz}^{medium}}\right). \tag{39}$$

It is now clear that if the medium is air, as it has been so far, $n_{THz}^{medium} = n_{THz}^{air} = 1$ and Eq. 39 does not have a real solution. Moreover, because of refraction at the air/silicon interface, even a THz beam incoming from the substrate leads to exactly the same result. Therefore, in order to find a solution to Eq. 39 it is necessary that the THz beam propagates through a material with a refractive index larger than the optical group index, yet without undergoing angular refraction.

For instance, we envision that this could be practically achieved by etching the rear facet of the substrate into a triangular shape that forms an angle complementary to α_{broad} (i.e., $\pi/2 - \alpha_{broad}$, being $\alpha_{broad} = 42.26°$ for the group index $n_g = 2.3$ utilized in previous calculations) at the corner with the stack of silicon dioxide and LN films, as depicted in the sketch of Fig. 21. This way, a THz beam inclined by the same angle α_{broad} would orthogonally hit the silicon/air interface, preventing angular refraction, and thus accumulating the correct amount of delay necessary to give rise to broadband detection with multiple resonating elements.

Supplementary References

[1] M. Peccianti, M. Clerici, A. Pasquazi, L. Caspani, S. P. Ho, F. Buccheri, J. Ali, A. Busacca, T. Ozaki, and R. Morandotti. "Exact reconstruction of thz Sub-λ source features in knife-edge measurements." IEEE Journal on Selected Topics in Quantum Electronics, **19**(1):97–107 (2013).

[2] M. A. De Araújo, R. Silva, E. De Lima, D. P. Pereira, and P. C. De Oliveira. "Measurement of Gaussian laser beam radius using the knife-edge technique: improvement on data analysis." Applied Optics, Vol. 48, Issue 2, pp. 393-396, **48**(2):393–396 (2009).

[3] Constantine A. Balanis. Antenna theory analysis and design 4th edition. Wiley (2016).
